# Glutamate 1-semialdehyde aminotransferase is connected to GluTR by GluTR-binding protein and contributes to the rate-limiting step of 5-aminolevulinic acid synthesis

**DOI:** 10.1093/plcell/koac237

**Published:** 2022-08-16

**Authors:** Neha Sinha, Jürgen Eirich, Iris Finkemeier, Bernhard Grimm

**Affiliations:** Institute of Biology/Plant Physiology, Humboldt-University Berlin, 10115 Berlin, Germany; Institute for Plant Biology and Biotechnology, Plant Physiology, Westfälische Wilhelms-Universität, 48149 Muenster, Germany; Institute for Plant Biology and Biotechnology, Plant Physiology, Westfälische Wilhelms-Universität, 48149 Muenster, Germany; Institute of Biology/Plant Physiology, Humboldt-University Berlin, 10115 Berlin, Germany

## Abstract

Tetrapyrroles play fundamental roles in crucial processes including photosynthesis, respiration, and catalysis. In plants, 5-aminolevulinic acid (ALA) is the common precursor of tetrapyrroles. ALA is synthesized from activated glutamate by the enzymes glutamyl-tRNA reductase (GluTR) and glutamate-1-semialdehyde aminotransferase (GSAAT). ALA synthesis is recognized as the rate-limiting step in this pathway. We aimed to explore the contribution of GSAAT to the control of ALA synthesis and the formation of a protein complex with GluTR. In *Arabidopsis thaliana*, two genes encode GSAAT isoforms: *GSA1* and *GSA2*. A comparison of two *GSA* knockout mutants with the wild-type revealed the correlation of reduced GSAAT activity and ALA-synthesizing capacity in leaves with lower chlorophyll content. Growth and green pigmentation were more severely impaired in *gsa2* than in *gsa1*, indicating the predominant role of GSAAT2 in ALA synthesis. Interestingly, GluTR accumulated to higher levels in *gsa2* than in the wild-type and was mainly associated with the plastid membrane. We propose that the GSAAT content modulates the amount of soluble GluTR available for ALA synthesis. Several different biochemical approaches revealed the GSAAT–GluTR interaction through the assistance of GluTR-binding protein (GBP). A modeled structure of the tripartite protein complex indicated that GBP mediates the stable association of GluTR and GSAAT for adequate ALA synthesis.

## Teaser

A mechanism is described that maintains adequate 5-aminolevulinic acid activity via a tripartite protein complex, representing the rate-limiting step in the synthesis of vital tetrapyrrole pigments.

## Introduction

5-Aminolevulinic acid (ALA) is the unique metabolic precursor for tetrapyrrole biosynthesis (TBS). Two different ALA-synthesizing pathways have evolved. In β-proteobacteria, fungi, and animals, ALA is synthesized from succinyl coenzyme A and glycine by ALA synthase (EC 2.3.1.37, Whiting and Granick, 1976). In other bacteria and photosynthetic eukaryotes, ALA synthesis requires two distinct enzymes and starts with glutamyl-tRNA. This activated glutamate is reduced by glutamyl-tRNA reductase (GluTR, EC 1.2.1.70, encoded by *HEMA*) to glutamate 1-semialdehyde (GSA), which is then transaminated by GSA aminotransferase (GSAAT, EC 5.4.3.8, encoded by *GSA*) to ALA (Mayer and Beale, 1990). While the ALA synthase of animals and fungi is restricted to mitochondria, plant ALA synthesis takes place in plastids. It is generally accepted that ALA synthesis is the rate-limiting step in TBS (Beator and Kloppstech, 1993; McCormac et al., 2001).

In plants, TBS leads to the biosynthesis of chlorophyll, heme, phytochromobilins, and siroheme. At the level of protoporphyrin IX (Proto), the pathway splits into two branches. Fe^2+^ chelation of Proto leads to protoheme, while the insertion of Mg^2+^ into Proto gives rise to Mg protoporphyrin, the precursor of chlorophylls. These essential photosynthetic pigments facilitate light absorbance and transfer excitation energy to the reaction centers of the two photosystems, where the special pair in the reaction centers provides electrons for the photosynthetic electron transport chain by charge separation. Heme binds various gases and plays vital roles in electron transfer and redox reactions (Tanaka and Tanaka, 2007; Yin and Bauer, 2013; Brzezowski et al., 2015). Moreover, some protoheme molecules are converted into phytochromobilin to contribute to light-dependent signaling. Siroheme derived from uroporphyrinogen is synthesized in a different branch of the pathway and acts as a redox cofactor in nitrite and sulfite reductases during nitrogen and sulfur assimilation, respectively (Bryant et al., 2020).

**Figure 1:**
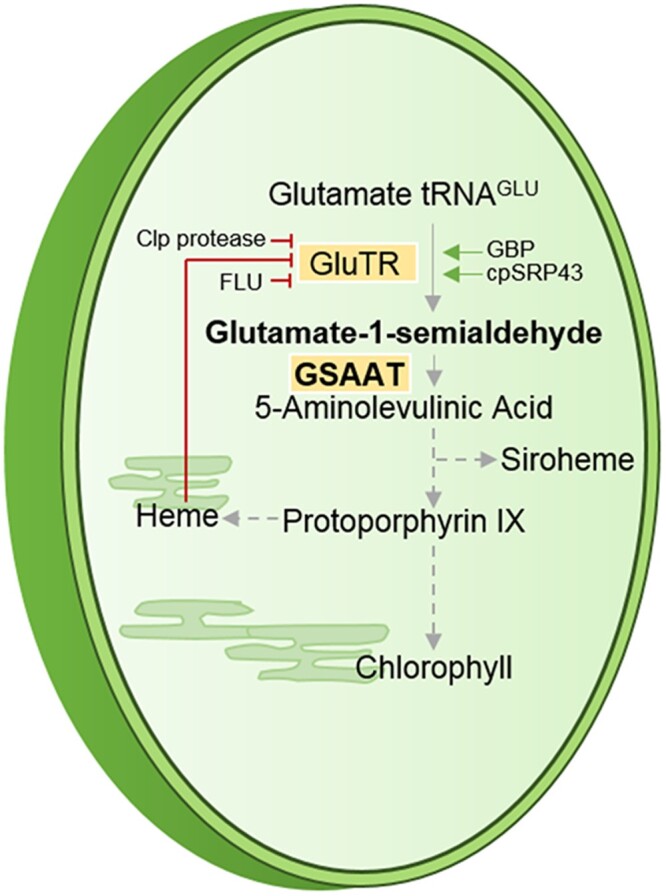
Schematic depiction of plastidic TBS starting with glutamyl-tRNA^Glu^ and leading to chlorophylls, heme, and siroheme with an emphasis on the enzymatic steps in ALA synthesis.

As ALA synthesis is considered to be the rate-limiting step in the TBS pathway (Beator and Kloppstech, 1993; McCormac et al., 2001), it is precisely controlled by multiple factors at both the transcriptional and posttranslational levels (Figure 1; Tanaka and Tanaka, 2007; Mochizuki et al., 2010, Brzezowski et al., 2015). GluTR appears to be the main target of regulatory and accessory factors. Due to the light-dependent activity of protochlorophyllide oxidoreductase (POR) in angiosperms, a fundamental suppression of light-induced chlorophyll biosynthesis occurs in angiosperms in the dark, which is mediated by fluorescent in blue light (FLU) to inactivate GluTR. FLU is a tetratricopeptide-repeat protein that assembles GluTR into a multicomponent complex at the thylakoid membrane, together with the downstream enzymes POR, Mg protoporphyrin monomethyl ester (MgProtoME), oxidative cyclase (MgP-Cys), and vinyl reductase (VR) (Meskauskiene et al., 2001; Kauss et al., 2012). This GluTR inactivation complex forms not only in the dark but also under adverse environmental conditions (Hou et al., 2019). Heme-dependent inactivation of ALA synthesis is induced by heme-binding of the GluTR-binding protein (GBP), which normally binds tightly to GluTR and protects it from degradation (Czarnecki et al., 2011; Richter et al., 2019). Heme-bound GBP dissociates from GluTR, which can subsequently be recognized by the selector protein ClpS and subsequently be degraded by the Clp protease system (Apitz et al., 2016; Richter et al., 2019). GluTR also interacts with the reductants thioredoxin and Nicotinamide adenine dinucleotide phosphate-dependent thioredoxin reductase C and is stabilized in the reduced form (Richter et al., 2013). Chloroplast signal recognition particle 43 (cpSRP43) acts as a chaperone for GluTR by preventing its aggregation (Wang et al., 2018).

Unlike the interactions of GluTR with these various regulatory and accessory proteins, the contribution of GSAAT to the control of ALA synthesis has not been well studied. The single *gsa* gene in the green alga *Chlamydomonas reinhardtii* was found to be induced by blue light via a Ca^2+^-mediated mechanism (Im et al., 1996). The metabolic effect of the repression of GSAAT expression was first documented for transgenic tobacco (*Nicotiana tabacum*) lines using a *GSA* antisense RNA approach (Höfgen et al., 1994; Härtel et al., 1997; Zavgorodnyaya et al., 1997), which was followed by reports on the impact of compromised GSAAT expression in rapeseed (*Brassica napus*) and tomato (*Solanum lycopersicum*) (Tsang et al., 2003; Lytovchenko et al., 2011). GSAAT deficiency was displayed by a variegated yellow-green phenotype, indicating a reduced chlorophyll content as a result of diminished ALA formation. Moreover, a point mutation in the rice (*Oryza sativa*) *GSA* gene also resulted in a yellow-leaf phenotype (Wang et al., 2021). However, posttranslational control mechanisms involving interacting protein partners of GSAAT have not yet been clarified.

Plant GSAATs belong to the pyridoxal phosphate (PLP)-dependent family of transaminases (Toney, 2014) and accumulate in the stroma of chloroplasts as a 46-kDa protein. These enzymes catalyze the transfer of the C2 amino group of GSA to the C1 position and have an absorption maximum specific for vitamin B6-dependent enzymes (Jahn et al., 1992). Spectral analysis of the reaction mechanism of the cyanobacterium *Synechococcus* PCC6301 enzyme revealed that transamination is initiated by the vitamin B6 derivative pyridoxamine phosphate (PMP; Smith et al., 1991). In two half-reactions, the conversion of GSA to ALA occurs preferentially via the intermediate diaminovalerate (DAVA), instead of dioxovalerate. Thus, in the first step of this reaction, PMP provides the amino group for the metabolic intermediate and is converted to PLP; in the second step, PMP is regenerated upon ALA formation (Smith et al., 1998; Stetefeld et al., 2006).

The structures of GSAAT from *Synechococcus* PCC6301 and *Arabidopsis thaliana* have been determined (Hennig et al., 1997; Song et al., 2016). GSAAT forms an asymmetric dimer, as the orientations of both cofactor binding and the gating-loop are asymmetrically disposed in the crystal structure. In one monomer, PMP is bound, with the gating loop fixed in the open state, while the other monomer binds either PMP or PLP and the gating loop is closed. This structural feature supports previous biochemical studies on the GSAAT reaction mechanism with DAVA as an intermediate (Smith et al., 1991; Song et al., 2016).

Based on the X-ray crystal structures of the archaeal GluTR and the cyanobacterial GSAAT (Hennig et al., 1997; Schubert et al., 2002), a complex consisting of the two dimeric enzymes has been modeled (Moser et al., 2001), which suggests that GSA is transferred directly from GluTR to GSAAT. The formation of a GluTR–GSAAT complex was later proven experimentally in both *Escherichia coli* (Lüer et al., 2005) and *C. reinhardtii* (Nogaj and Beale, 2005). Moreover, a HemA–HemL complex was also shown by co-purification of these proteins from *Acinetobacter baumannii* (Nardella et al., 2019).

These findings and many others suggest that enzymes belonging to the same metabolic pathway are likely to be organized in protein complexes in subcellular compartments instead of being randomly distributed within them. However, how GluTR supplies the substrate for the next enzyme GSAAT remains an intriguing question in the control of ALA synthesis in plants (Lüer et al., 2005; Nogaj and Beale, 2005). So far, no GluTR–GSAAT complex has been detected in plants and, surprisingly, a physical interaction between GluTR and GSAAT has still not been confirmed in plants.

The Arabidopsis genome contains two homologous genes encoding GSAAT, *AtGSA1* and *AtGSA2*. However, apart from the evolutionary significance of this gene duplication, the impact of the expression of both *GSA* genes in space and time, and in response to changing environmental conditions, is unclear. Using null mutants of each of the *GSA* genes, we set out to define the functions of the two GSAAT isoforms in ALA biosynthesis, and in particular their roles in the possible relationship with GluTR. We applied several experimental approaches to characterize the interaction of GSAAT and GluTR. We demonstrate that only GBP mediates the formation of a complex between GSAAT and GluTR.

## Results

### Two genes encode isoforms of GSAAT in Arabidopsis

The two isoforms of GSAAT in Arabidopsis share 90% sequence identity. As indicated in the phylogenetic tree (Supplemental Figure S1A), the high similarity between these isoforms points to a recent gene duplication. Based on publicly available expression data (Klepikova et al., 2016), *GSA1* and *GSA2* are sometimes expressed at similar levels in different organs and at different developmental stages. However, divergent expression was found in roots, seeds, and leaves during the development of light-exposed seedlings. Thus, *GSA1* is preferentially expressed in dry seeds, roots, and senescent leaves, while *GSA2* is predominantly expressed in green leaves and shoots (Supplemental Figure S1B). This expression pattern indicates that *GSA2* plays an especially important role during the development of aerial green organs.

To assess the relative physiological significance of the two GSAAT isoforms during the daily course of light-induced ALA synthesis in leaves, we quantified transcripts of *GSA1*, *GSA2*, and *HEMA1* (which encodes the more dominant of the two GluTR isoforms) in the leaves of four-week-old seedlings grown under short-day (SD) conditions (10 h light [120 µmol photons m^−2^ s^−1^]/14-h dark) via reverse transcription–quantitative polymerase chain reaction. The results revealed the diurnal oscillation of the levels of transcripts of all three genes, with a rise in the second half of the dark period and a peak 2 h after the onset of illumination (Supplemental Figure S2A). This diurnal oscillation, with the light-dependent accumulation of *GSA* and *HEMA* transcripts in *A. thaliana*, resembles the previously reported expression patterns of the single-copy *GSA* and *HEMA* genes in *Chlamydomonas* (Nogaj and Beale, 2005).

**Figure 2:**
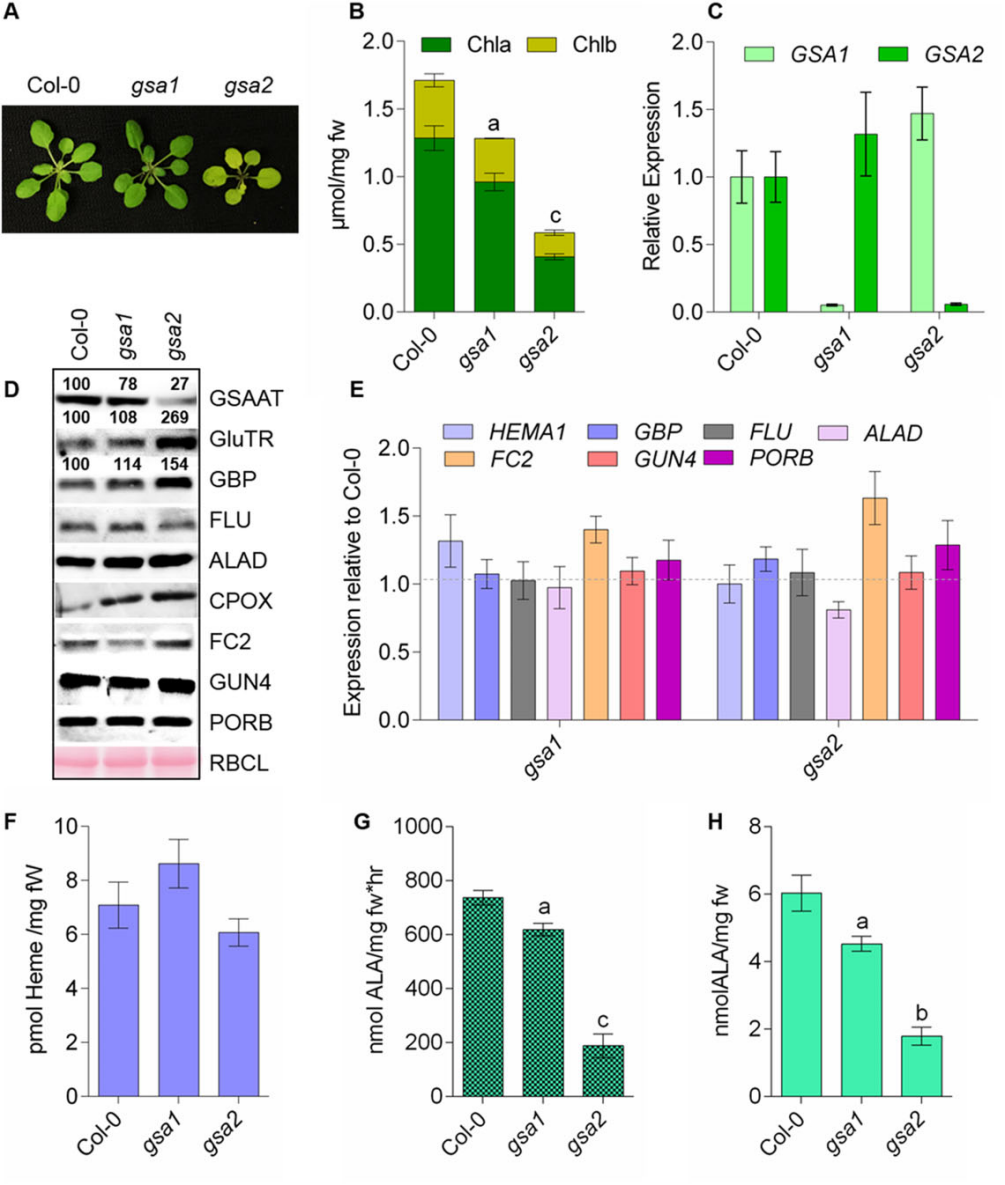
Analysis of *gsa1* and *gsa2* mutants grown under standard light intensity (120 µmol photons m^−2^ s^−1^) in SD. A, Representative 4-week-old seedlings of the wild-type (Col-0) and mutant T-DNA insertion lines *gsa1* and *gsa2*. B, Comparison of their chlorophyll *a* and *b* contents. C, Relative expression levels of *GSA1* and *GSA2* in leaves of 4-week-old seedlings of the three genotypes. The leaf material was harvested 4 h after the transition from dark to light. (D) Immunoblot analysis shows the levels of proteins involved in ALA and TBS in wild-type and the two *gsa* mutants. The samples were fractionated on 12% SDS gels. The numbers between the immunoblots represent normalized abundancies of GSAAT, GluTR, and GBP in the examined genotypes relative to the control seedlings (Col-0) using three immunoblot replicates. The names of the targeted proteins are indicated on the right. The Ponceau-stained large subunit of RuBisCO (RBCL) served as a loading control. ALAD, ALA dehydratase. E, Transcript levels of the indicated genes encoding enzymes involved in ALA and TBS; *HEMA1*, GluTR. F, Heme content, (G) GSAAT activity, and (H) ALA-synthesizing capacity in detached leaves of 4-week-old wild-type, *gsa1* and *gsa2* seedlings grown under SD conditions. Data in (B), (G), and (H) are given as the mean of the standard deviation of biological replicates from three different harvests. Statistical significance compared with Col-0 seedlings is indicated by ^a^*P* ≤ 0.05, ^b^*P* ≤ 0.01, ^c^*P* ≤ 0.001 using Student’s *t* test in the Supplemental Data Set S1; fw, fresh weight.

Of the two enzymes that contribute to ALA synthesis in leaf extracts, GluTR became most abundant 4–6 h after the transition from dark to light (between 12:00 and 14:00), while the GSAAT content remained stable over the entire 24-h photoperiod (Supplemental Figure S2B). This observation points to a balance between de novo synthesis and degradation of GSAAT, despite the diurnal oscillation of *GSA* transcript levels (Grimm, 1990). Using an antibody against recombinant Synechococcus GSAAT (Grimm, 1990), one immunoreactive GSAAT band was detected after sodium dodecyl-sulfate (SDS)–polyacrylamide gel electrophoresis (PAGE). The two single *GSA* knockout mutants (see next paragraph) displayed a single 46-kDa immunoreactive band, indicating that the two isoforms showed the same mobility and that the immune signal corresponded to the combined amounts of both isoforms.

### The physiological consequences of GSA knockout mutations

To analyze the functional significance of each of the two GSAAT isoforms, we compared knockout mutants for each of the two *GSA* genes to wild-type controls (Col-0). Four-week-old seedlings were grown under standard SD conditions as described above (Figure 2A). *GSA1* compensated less effectively for the loss of *GSA2* than did *GSA2* for the lack of *GSA1* (Figure 2B). Thus, the *gsa2* mutant showed a more marked (66%) decrease in chlorophyll a/b content compared to the wild-type (Figure 2B). By contrast, the growth rates and chlorophyll contents of *gsa1* seedlings were similar to wild-type levels. In both *gsa* knockout mutants, the transcript levels of the intact *GSA* isogene were not significantly altered compared to wild-type levels (Figure 2C). We compared the contents of GSAAT and other enzymes involved in ALA synthesis with the corresponding transcript levels in the wild-type and the two mutants (Figure 2, D and E). Compared to wild-type, the leaf extracts of the two *gsa* mutants always displayed lower levels of GSAAT encoded by the intact *GSA* gene. These results indicate that both *GSA* genes contribute to the total GSAAT content in leaves, but a deficient *GSA* gene is only partially compensated for by expression of the second *GSA* gene (Figure 2C). The lowest GSAAT content was found in *gsa2* leaf extract. Interestingly, *gsa2* leaf extracts contained higher GluTR and GBP levels than the other genotypes. Despite the variation in GluTR content, the transcript levels of *HEMA* and other representative genes involved in ALA and TBS were always similar in the wild-type and the two *gsa* mutants (Figure 2E).

Despite the reduced chlorophyll content in the two *gsa* mutants relative to wild-type, the heme content in leaves was not significantly altered compared to wild-type levels (Figure 2F). Moreover, the in-vitro GSAAT activity of plant extracts and the in vivo rate of ALA synthesis in leaves, which accounts for overall ALA synthesis by the enzymes GluTR and GSAAT, correlated with the decreases in GSAAT contents of the two *gsa* lines relative to wild-type (Figure 2, G and H). Compared to *gsa1*, *gsa2* leaves showed significantly reduced GSAAT activity and as result, a marked loss of chlorophyll content (Figure 2, A and B), indicating that GSA2 has a greater effect on chlorophyll accumulation than GSA1. However, it is obvious that both GSAAT isoforms are required for the biosynthesis of adequate ALA levels in chlorophyll-synthesizing leaves. While *gsa2* leaves showed strongly reduced GSAAT activity, as indicated by the marked loss of chlorophyll biosynthesis in leaves (Figure 2, A and B), *gsa1* exhibited a less pronounced reduction in GSAAT activity and chlorophyll content. Thus, loss of the *GSA2* gene has a greater effect on chlorophyll accumulation than knockout of *GSA1*, but both GSAAT isoforms are required for the biosynthesis of adequate ALA levels in chlorophyll-synthesizing leaves.

### Complementation of *gsa2* via *GSA1* expression

**Figure 3:**
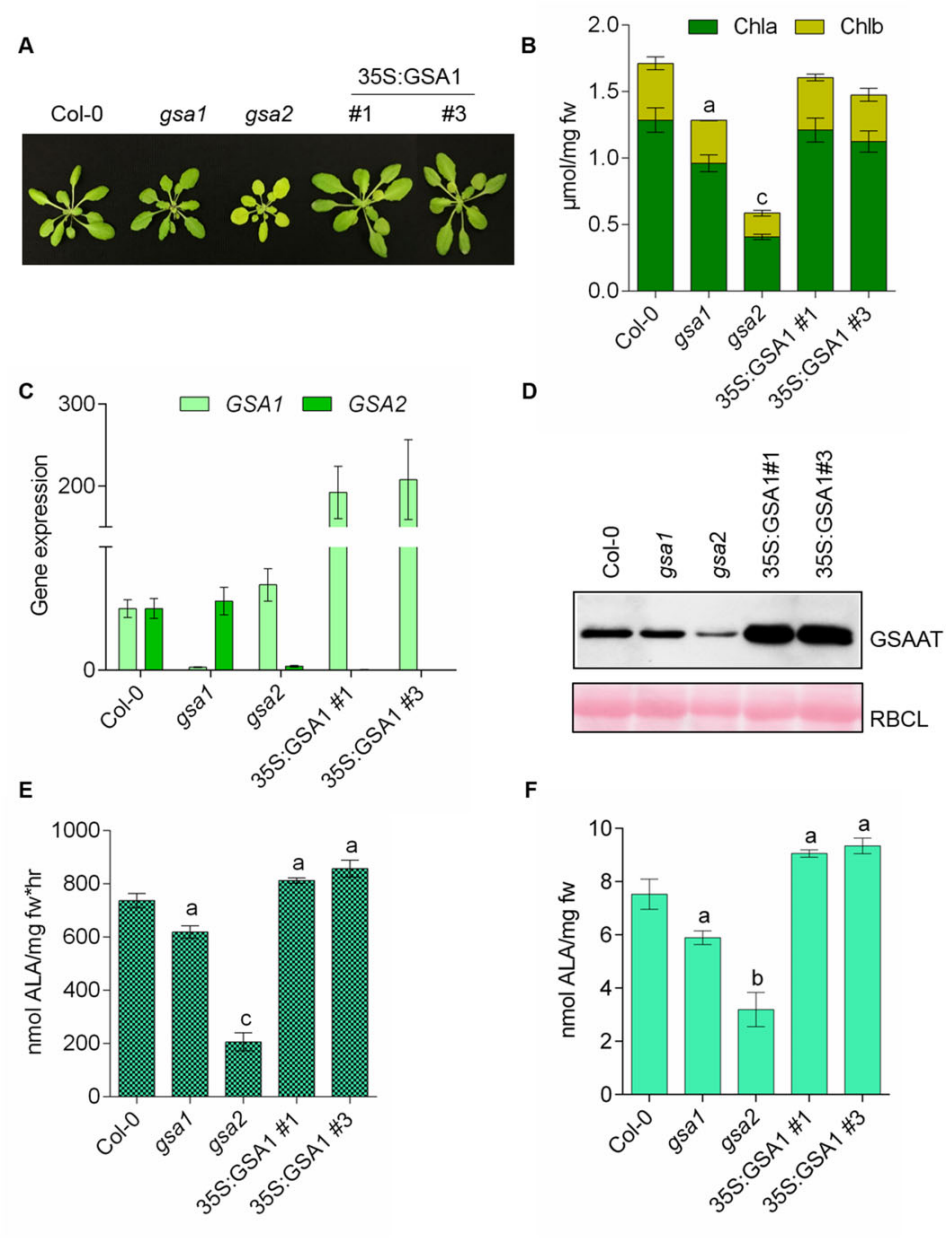
Complementation of the *gsa2* mutant by constitutive expression of *p35S:GSA1*. A, Comparison of 4-week-old SD-grown seedlings of wild-type (Col-0) *gsa1, gsa2* and the two *gsa2* lines #1 and #3 expressing *35S*:*GSA1.* B, Chlorophyll *a* (dark green, bottom) and *b* (yellow-green, top) contents. C, *GSA1* (light green) and *GSA2* (dark green) transcript levels. D, Immunoblot analysis of the indicated proteins. RBCL stained with Ponceau red is shown as a loading control. E, GSAAT enzyme activity. F, Relative rates of ALA synthesis in the indicated lines. Leaf material was harvested 4 h after the onset of light from 4-week-old seedlings grown under standard SD conditions. All the data in (B), (E), and (F) are provided as a mean of the standard deviation of biological replicates from three different harvests. Statistical significance compared with Col-0 seedlings was performed by ^a^*P* ≤ 0.05, ^b^*P* ≤ 0.01, ^c^*P* ≤ 0.001 using Student’s *t* test and is found in Supplemental Data Set S1; fw, fresh weight.

Deletion of *GSA2* caused a more severe chlorophyll-deficient phenotype in seedlings than the lack of GSAAT1. We therefore asked whether the *gsa2* mutant could be rescued by overexpression of *GSAAT*. We analyzed two transgenic lines (T3 generation) expressing *GSA1* under the control of the 35S promoter (35S:GSA1#1, 35S:GSA1#3) from two independent transformations. The transgenic lines in the *gsa2* background were macroscopically indistinguishable from wild-type seedlings and accumulated similar chlorophyll contents to wild-type (Figure 3, A and B). This could be attributed to the enhanced amounts of *GSA1* transcripts and the correspondingly high accumulation of GSAAT1 (Figure 3, C and D). The GSAAT activity of leaf extracts and the capacity for ALA synthesis in leaf discs were not significantly higher than those of wild-type seedlings in these complementation experiments (Figure 3, E and F). We therefore conclude that the loss of *GSA2* could indeed be fully rescued by overexpressing the *GSA1* transgene.

### Impact of different light intensities on ALA synthesis and the subplastidal localization of ALA biosynthesis enzymes

**Figure 4:**
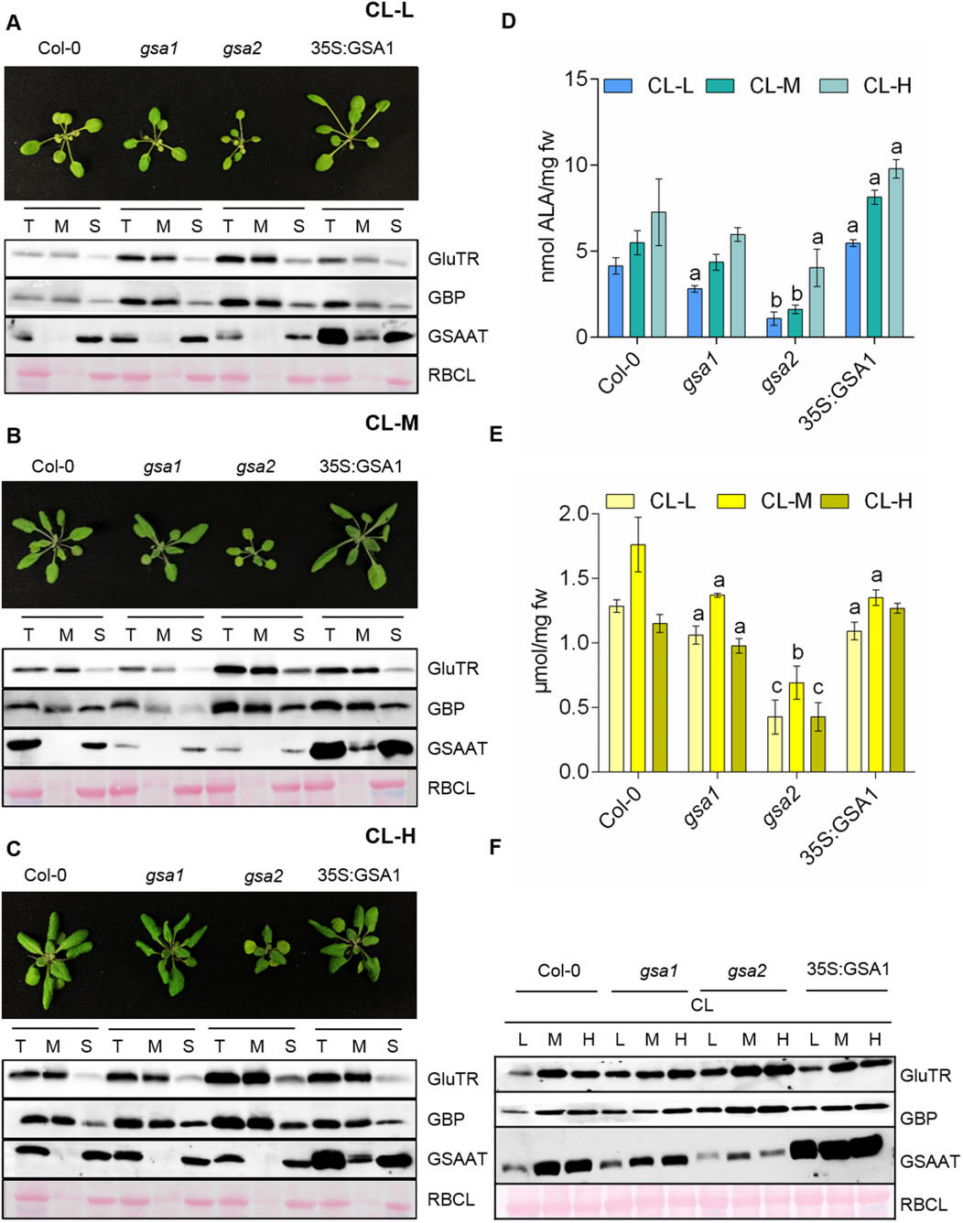
Analysis of the wild-type, *gsa1, gsa2* and *35S:GSA1(gsa2)* lines under different light intensities. A–C, Top, 4-week-old seedlings of all four genotypes grown in CL under standard (CL-M; 120 µmol photons m^−2^ s^−1^), low (CL-L; 10 µmol photons m^−2^ s^−1^), and high light levels (CL-H; 300 µmol photons m^−2^ s^−1^). Bottom, immunoblot analysis of GluTR, GBP, and GSAAT in crude plastid extract (T), membrane (M), and stroma (S) fractions. (D) GSAAT activity in total leaf extracts from the four indicated genotypes exposed to the three different light intensities, CL-L, CL-M, and CL-H. E, Chlorophyll *a* and *b* contents. For (D) and (E), data represent the standard error of biological replicates from three different harvests. The statistical significance was performed using Student’s *t* test, when ^a^*P* ≤ 0.05, ^b^*P* ≤ 0.01, and ^c^*P* ≤ 0.001 mentioned in Supplemental Data Set S1; fw, fresh weight. F, Immunoblot analysis of the indicated proteins in each of the four genotypes under CL in all three light intensities (L, low, M medium/standard, H high). In (A–C) and (F), the Ponceau-stained large subunit of RuBisCO (RBCL) served as a loading control.

We aimed to verify the impact of each of the *GSA* genes on ALA synthesis in 4-week-old seedlings of the two *gsa* mutants relative to a *GSA1*-overexpressing line and the wild-type under different light intensities. The seedlings were initially grown under standard continuous light (CL-M, 120 µmol photons m^−2^ s^−1^) before being exposed to three different light intensities. The seedlings were grown under CL at either standard light intensities (CL-M) or low light intensity (CL-L, 10 µmol photons m^−2^ s^−1^ for 1 week) and high light intensity (CL-H, 300 µmol photons m^−2^ s^−1^ for 3 days) before leaf samples were harvested (Figure 4, A–C). We compared the GSAAT content with the GluTR and GBP contents in stroma and membrane fractions and analyzed GSAAT activity and chlorophyll content in response to different light intensities in different genotypes (Figure 4, D and E). GSAAT was always found in the stromal fraction, while it was also detected in the membrane fraction of the GSA1-overexpressor line. The *gsa2* lines accumulated larger amounts of GSAAT under CL-M than the respective CL-H- and CL-L-grown samples, although the level of GSAAT in *gsa2* amounted to only one-third that of the wild-type (Figure 4F). The constitutive expression of *GSA1* in the *35S:GSA1* line resulted in similar amounts of GSAAT under all three light intensities, whereas *gsa1* contained more GSAAT when grown under CL-H versus CL-M conditions. Thus, besides the differences in the total amounts of GSAAT between the two *gsa* mutants, the relative levels of GSAAT varied in each mutant depending on the light intensity (Figure 4F). Compared to the wild-type, the *gsa* mutants always contained lower levels of GSAAT and the *GSA1*-overexpressing line showed higher GSAAT activity in leaf extracts (Figure 4D). Overall, an association between GSAAT activity and chlorophyll content can be discerned: low light intensity was associated with the lowest chlorophyll contents, as well as low GSAAT contents and enzyme activity, in all genotypes examined.

Strikingly, in the wild-type, GSAAT-deficient, and *GSA1-*overexpressing mutant lines, GluTR and GBP contents were higher in the membrane fraction than in the stromal fraction under all three light conditions. The ratios of GluTR to GBP in the membrane and stromal fractions remained similar in all lines and under all light intensities. GluTR and GBP accumulated to a high extent in the *gsa2* mutant.

These findings in SD- and CL-grown seedlings (Figures 2 and 4) underline the critical importance of GSAAT for ALA synthesis. The phenotypes of the two *gsa* mutants and *35S:GSA1* line confirm the finding that the impact of *GSA2* deficiency is more pronounced than that of *GSA1*. *GSA2* acts predominantly on ALA synthesis in leaves, as GSAAT2 deficiency was associated with the retarded growth of seedlings, low chlorophyll accumulation, reduced GSAAT activity, and a low ALA synthesis rate—phenotypes that were not observed in GSAAT1-deficient seedlings. Loss of GSAAT2 had the greatest impact on ALA synthesis, although GluTR accumulated to higher levels in *gsa2* than in wild-type plants. These observations suggest that GSAAT contributes to the rate-limiting capacities of ALA biosynthesis in planta: the amount of GSAAT defines the quantity of GluTR promoting ALA biosynthesis.

### GSAAT stability during heme-dependent inactivation of ALA biosynthesis

We recently demonstrated the heme-dependent posttranslational control of GluTR degradation in Arabidopsis (Richter et al., 2019). In the absence of free heme, GBP is bound to GluTR, but the complex dissociates upon binding of heme to GBP. Soluble GluTR is susceptible to proteolysis by the Clp protease system, while membrane-bound GluTR is protected from degradation (Richter et al., 2019). Considering the elevated GluTR content in the *gsa2* mutant, we asked whether an excessive accumulation of heme might also affect the stability of GSAAT. To examine the potential heme-dependent turnover of GSAAT, we incubated leaves of the two *gsa* mutants, the *35S:GSA1* line, and the wild-type with and without an exogenous supply of ALA in the dark and measured the levels of ALA-synthesizing proteins in the soluble and membrane-associated fractions of total leaf extracts (Supplemental Figure S3, A–C).

In wild-type leaf samples, the reduction in GluTR content was confirmed and, upon supplementation with ALA, the enzyme was found to be below the detection limit after 24 h in the dark. Like in the wild-type, despite the addition of exogenous ALA to the *gsa1* mutant and the *GSA1*-overexpressing *gsa2* line in the dark, the levels of GluTR in membrane and stromal fraction were clearly reduced compared to incubation in mock solution (Supplemental Figure S3, A–C). In contrast to the wild-type, GluTR proteolysis was attenuated in *gsa2*, and the GluTR content of this mutant was higher in the membrane fraction than the soluble fraction. Thus, the lack of GSAAT2 stimulates the association of GluTR with the membrane (Supplemental Figure S3D).

However, a 24-h incubation in the presence of exogenous ALA did not result in significant degradation of GSAAT in either the wild-type or any other genotypes (*gsa1*, *gsa2*, and *35S:GSA1* line). Furthermore, the FLU contents in the membrane fractions of all leaf samples remained stable. The amounts of GBP in the soluble and membrane fractions of the plant samples resembled those of GluTR, that is, lower contents in ALA-treated wild-type, *gsa1* and the *35S:GSA1* plants compared to plants incubated in the dark without ALA supply. The stability of GBP appeared to be slightly elevated in the membrane fraction of *gsa2*.

In light of the reduced amounts of chlorophyll in the *gsa* mutants, we examined the effects of lower GSAAT content on photosynthetic capacity and the assembly of the photosystems. Normalized to chlorophyll content, the *gsa* mutants showed the same amounts of fully assembled photosynthetic complexes as the wild-type (Supplemental Figure S4A). Keeping in mind the reduced chlorophyll content in *gsa1* and *gsa2* (16% and 66% of wild-type chlorophyll content, respectively), the levels of proteins of photosystems I and II and their antennas were always reduced when equal amounts of protein from each extract were analyzed by SDS–PAGE and subsequent immunoblotting analysis (Supplemental Figure S4B). Although the *gsa* mutants are chlorophyll deficient, they nevertheless assembled the complete photosynthetic complexes under standard growth conditions. Thus, the formation of complete photosynthetic units was not impaired by GSAAT deficiency and the resulting reduction in chlorophyll accumulation during SD growth. These findings agree with results from tobacco *GSA* antisense plants (Höfgen et al., 1994; Härtel et al., 1997).

### Interaction between GSAAT and GluTR

**Figure 5:**
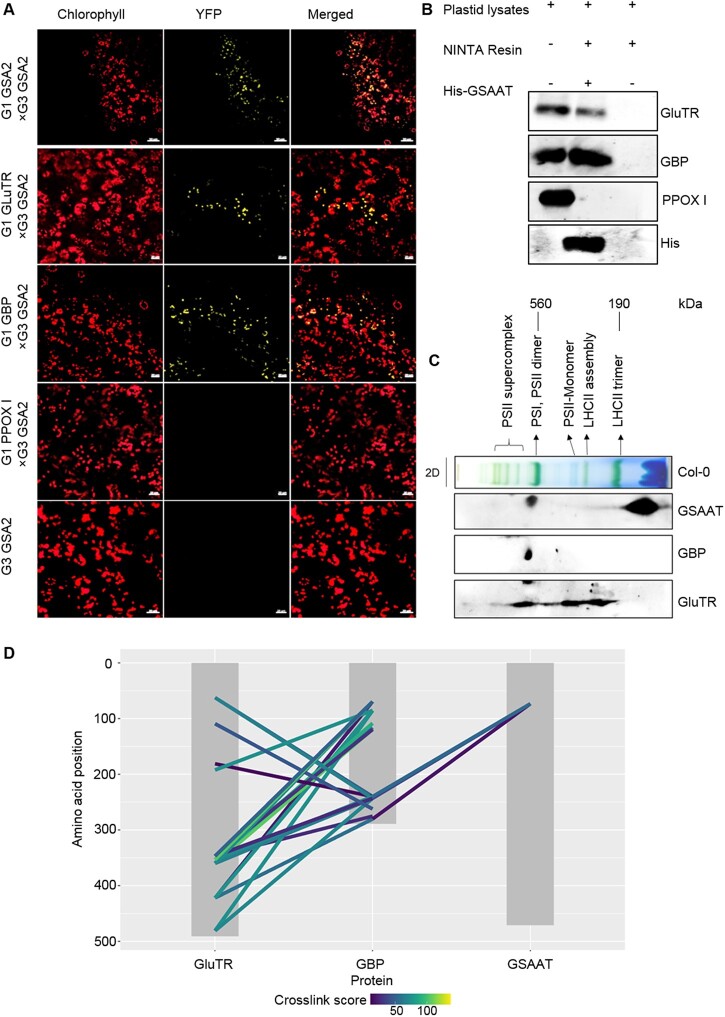
Physical interaction of GSAAT with both GluTR and GBP. A, Visualization of protein interactions in chloroplasts by BiFC assays. Images show *N. benthamiana* cells transiently expressing constructs encoding the YFP fusion proteins (indicated by yellow fluorescence). Red fluorescence represents chloroplast autofluorescence, and overlay images show merged signals. Each image is representative of more than three replicates. BiFC assays demonstrate dimerization of GSAAT2 (top row), and interaction of GSAAT2 with GluTR (second row) and GBP (second row). Co-expression of GSAAT-YFPc and either GBP-YFPn, HEMA1-YFPn, or GSAAT-YFPn is presented. Scale bars 20 µm. B, In vitro His-tagged pull-down experiment with purified wild-type Arabidopsis plastid extracts and recombinant 6× His-GSAAT as bait protein demonstrates that GSAAT binds to GluTR and GBP. Proteins were immunologically detected in the eluate obtained after release from bound GSAAT. C, Distribution of GSAAT, GluTR, and GBP in different plastid protein complexes. Solubilized wild-type plastid protein complexes were fractionated by BN–PAGE in the first dimension, and SDS–PAGE analysis in the second. The proteins were detected using the indicated specific antibodies. D, Visualization of cross-links between the three proteins of interest. Grey boxes represent the protein sequences. Colored lines represent cross-links connecting an amino-acid position (*y*-axis) with a peptide from another protein. Colored according to the MQ cross-link score.

A physical interaction between GluTR and GSAAT was demonstrated using several different approaches in planta and in vitro. We conducted a bimolecular fluorescence assay in *Nicotiana benthamiana* leaf cells by transiently expressing gene constructs encoding complementary halves of yellow fluorescent protein (YFP) fused to each of the enzymes (Figure 5A). In addition to confirming that GSAAT2 forms dimers, this strategy also showed that GSAAT2 interacted with GluTR and GBP. To further confirm these interactions, we performed pull-down experiments on wild-type plastid lysates, using purified recombinant His-GSAAT as bait. Proteins bound to 6xHis-GSAAT were eluted with elution buffer containing 250-mM imidazole. Proteins of the eluate were detected by immunoblotting using antibodies against GluTR, GBP, and GSAAT (Figure 5B).

We also performed two-dimensional blue native–PAGE (2D BN–PAGE) of solubilized chloroplast protein extracts and probed the immunoblots with antibodies against GSAAT, GluTR, and GBP. This revealed that portions of GluTR, GSAAT, and GBP co-migrated in at least one protein complex with a molecular mass of ∼540 KDa (i.e. in the same range as the PSI, PSII dimer complex, Figure 5C). Thus, a certain proportion of GSAAT stably interacts with GluTR and GBP in an oligomeric protein complex.

To verify this finding using an alternative approach, we conducted a cross-linking mass spectrometry experiment with bilateral and trilateral combinations of the three recombinant proteins (GSAAT, GluTR, and GBP). Apart from the known GBP–GluTR interaction, we carried out combined assays for mutual interactions of GSAAT with either GluTR or GBP, followed by a cross-linking experiment with all three proteins. After cleaving the proteins with trypsin, we analyzed the resulting fragments by mass spectrometry to identify the cross-linked peptides of the three proteins in all possible dual and triple combinations. This approach allowed us to characterize the relationships among all three proteins.

**Figure 6:**
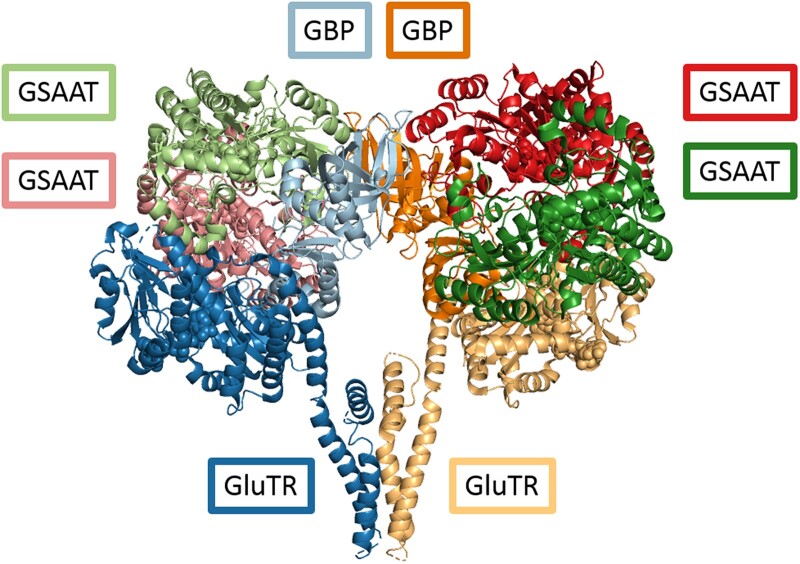
Model of the multimeric ALA-synthesizing complex. Superimposition of the heterodimeric complex comprising GluTR (yellow and dark blue, bottom middle) and GBP (light blue and orange, top middle), with a GSAAT (light and pink (top left) as well as dark green and dark red (top right)), based on molecular docking guided by cross-links identified by MS.

Multiple interactions between GBP and GluTR were detected, which confirmed previous findings of the GBP/GluTR interaction (Apitz et al., 2016; Song et al., 2016). Using a strict assessment of significantly stable bilateral interactions among the three proteins, no such interactions between GSAAT and either GluTR or GBP were detected by this approach. However, incubation of all three proteins together resulted in the formation of multiple physical cross-links between GBP and GSAAT, as well as GluTR. These interactions indicate the formation of an interdependent protein complex consisting of all three proteins (Figure 5D). The tightly cross-linked peptides of the proteins (based on cross-link scores) are summarized in Supplemental Table S1. We then compared the published structures of Arabidopsis GluTR, GSAAT, and GBP with the locations of cross-linked areas. This analysis indicated that GBP mediates the binding of GSAAT to the complex, as GSAAT was found to occupy some accessible areas of GBP in the complex. This in turn brings GSAAT into close proximity to GluTR (Figure 6). Thus, the combined application of all three proteins not only verified the interacting areas of GluTR and GBP, but it also revealed additional interaction sites of GBP and GSAAT. As indicated by the cross-linking experiments and by 2D BN–PAGE (Figures 5D and 6), this multimeric complex characterizes GBP as a scaffold protein. We therefore propose that efficient ALA synthesis from activated glutamate is facilitated by the formation of this complex, in which GBP brings GluTR and GSAAT into close proximity with each other.

We then performed binding studies with the proteins of the ALA synthesis complex to demonstrate the valuable support of GBP for the close contact between GluTR and GBP. We used purified GluTR as bait in an in vivo pull-down experiment using total plastid lysates from wild-type seedlings, a *gbp* knockout mutant (GBP KO), and a 35S:GSA1 line to demonstrate the mutual interdependence of GSAAT and GluTR on GBP. Proteins bound to GluTR were eluted with a buffer containing reduced glutathione. Proteins in the eluate were detected by immunoblotting using GSAAT- and GBP-specific antibodies, respectively (Supplemental Figure S5). Apart from the confirmation of the known positive control of the GluTR-GBP interaction (as initially shown in Czarnecki et al., 2011), interacting GSAAT was also detected in different amounts in the eluates. After its release from bound GST-tagged GluTR, a very small amount of GSAAT was detected in the eluate from total GBP KO extracts compared to that of the wild-type or the GSA1-overexpressor line. In summary, the physical association of GluTR and GSAAT during ALA synthesis is facilitated and intensified by GBP.

The strength of protein binding of this ALA synthesis complex was also verified by incubating recombinant GluTR with GSAAT alone or in combination with GBP and GSAAT. The interactions of these proteins in this assay were visible after thorough washing and elution of the bound proteins, which were immunologically identified in an SDS gel (Supplemental Figure S6, A and B). It turns out that GluTR did not directly pull-down GSAAT, but GSAAT was bound to GluTR through the assistance of GBP in the protein complex. Thus, GBP forms a scaffold complex for GSAAT and GluTR

A final approach helped us assess the mutual binding affinities of the proteins involved in the ALA–synthesis complex. We aimed to determine the strength of the pairwise interactions among GluTR, GSAAT and GBP and to assess the extent to which the complex with all three proteins is stabilized. This experiment revealed the strength of interactions between the GluTR, GSAAT, and GBP proteins. When 6xHis-GSAAT was used as a bait protein, the bilateral interaction to 6xHis-GluTR exhibited a *K*_D_ value of only 3 µM. Subsequently, we checked if the interaction between GSAAT and GluTR is strengthened in the presence of GBP. When GBP was labeled (as a bait), incubated with GluTR, and titrated with GSAAT, the interaction was tighter compared to the former bilateral interaction between GSAAT and GluTR. The *K*_D_ value of this interaction was 940 nM. The tightest association was observed between GluTR and GBP, with a *K*_D_ value of 42 nM, followed by the interaction of GBP with GSAAT, with a *K*_D_ value of 178 nM. The assay showed the strongest interaction with GBP and GluTR, and all three proteins revealed a more stable affinity than that of the GluTR–GSAAT complex (Supplemental Figure S7 and Supplemental Table S2).

## Discussion

### The implications of two genes encoding GSAAT

Like several other genes involved in TBS in *A. thaliana*, such as *HEMA*, *ALAD*, *CHLI* (encoding an Mg chelatase subunit), *PPOX* (protoporphyrinogen oxidase), and *FC* (ferrochelatase), there are two genes for GSAAT. Over the last decades, extensive research has been undertaken to understand the evolutionary significance of polyploidy (Van de Peer et al., 2017, 2021). Less attention has been paid to the impact of gene duplication on the diversification of metabolic pathways, the developmental program, stress responses, and overall viability (Van de Peer et al., 2021). Apart from divergent expression patterns of two paralogous genes, distinct functional impacts of the encoded isoforms can be expected. Notably, the paralogous genes involved in TBS are always derived from a single gene that was present in the plastidal ancestor, not from each of the two different organisms in the endosymbiosis that gave rise to photosynthetic eukaryotes (Oborník and Green, 2005).

The specific functions of the *HEMA, FC*, and *PPOX* paralogs and their encoded proteins have been examined (Bougri and Grimm, 1996; Lermontova et al., 1997; Masuda et al., 2003). The two isoforms of FC and PPOX act in different cellular sub-compartments (Che et al., 2000; Suzuki et al., 2002). These studies highlight the metabolic evolution of the plant TBS pathway, which was facilitated by the respective genomic signatures of genes for specific enzymatic steps, and they point to specialized metabolic and subcellular functions of such paralogs. It has become clear that, in these cases, one of the paralogs, often the dominant representative, is induced by light and expressed in an organ-specific fashion (mainly in source organs such as leaves, for example *HEMA1*, ferrochelatase [*FC2*], and *PPOX1*), while the second gene is expressed continuously, but at low levels, in non-photosynthetic tissues (sink organs, e.g. *HEMA2*, *FC1*, and *PPOX2*). Moreover, *FC1* was found to be specifically responsive to stress (Singh et al., 2002). Thus, the two isoforms of FC and PPOX are localized either in plastids and mitochondria, or in two different subplastidal compartments—the envelope and thylakoid membranes (Watanabe et al., 2001), to ensure the diverse allocation of their catalytic products inside plastids or heme-dependent enzymes inside and outside of plastids (Fan et al., 2019).

According to publicly available data (http://bar.utoronto.ca/efp/cgi-bin/efpWeb.cgi?primaryGene=AT5G63570&dataSource=Klepikova_Atlas&modeInput=Absolute.), the expression profiles of the two *GSA* genes generally do not differ very widely (Supplemental Figure S1B). In aerial green tissues during plant development up to senescence, *GSA2* is more highly expressed than *GSA1*. The reason for the maintenance of both *GSA* copies is still not clear. Based on the available expression patterns, *GSA2* is predominantly expressed in photosynthetic source tissue, while *GSA1* is preferentially expressed in sink organs (senescent leaves, roots, seeds). The overexpression of *GSA1* fully complemented the loss of *GSA2* in green tissues, indicating that the two enzymes are likely functionally equivalent. Further biochemical studies with purified GSSAT isoforms may confirm their specific activities and catalytic velocity.

It was previously proposed that two ALA pools (Huang and Castelfranco, 1990) are present to allocate ALA for heme or chlorophyll synthesis or to supply tetrapyrrole end products for tetrapyrrole-dependent proteins inside or outside of plastids. Currently, the localization of both GSAAT isoforms can only be assigned to the stroma of chloroplasts. However, a certain amount of the overproduced GSAAT1 in *gsa2* was associated with the plastid membrane (Figure 4, A–C). This fraction might represent an inactive proportion of GSAAT. In support of this hypotheses, GluTR was detected in both the stroma and membrane-associated fractions in all Arabidopsis genotypes analyzed (Schmied et al., 2018). These two fractions of GluTR represent an active form and an inactive, FLU-associated form, respectively, rather than two distinct active pools that contribute to different branches of TBS. We cannot exclude the possibility that rate-limiting and decisive metabolic steps are also controlled by the separation of well-defined pools of active proteins and a small amount of deposited proteins.

All current ideas on the organization of TBS are based on observations in the chloroplasts of young seedlings, in which ALA synthesis is expected to be localized to the stroma. Future studies of plastids in roots and other organs, such as the developing embryo, seed, or flower, are required to unravel the potential functional diversity of the isoforms in TBS. However, even if it is assumed that the occurrence of two *GSA* genes is based on two distinct versions of glutamate-directed ALA synthesis, we currently do not have evidence for biochemically specific but metabolically diverse functions of the two GSA variants in TBS. The observation that the lack of GSAAT2 leads to a higher accumulation of GluTR, which is mainly associated with the plastid membrane, points to the possible GSAAT-dependent regulation of GluTR levels during stromal ALA biosynthesis in green leaves. Only soluble GSAAT and GluTR contribute to ALA synthesis, while excess GluTR is stored on the membrane (see below). Nevertheless, both GSA isogenes are needed for adequate ALA synthesis, and the loss of either one results in chlorophyll deficiency, while the overproduction of GSAAT1 compensates fully for the loss of the *GSA2* gene.

### The contributions of the two GSAATs to the rate-limiting step in ALA synthesis

Proteostasis is defined as the dynamically controlled maintenance of a functional and coordinated proteome. To match the activity of the proteome for ALA and chlorophyll synthesis, the expression and availability of the required proteins are controlled and coordinated at different levels, including protein synthesis, targeting to plastids and translocation into subplastidal compartments, posttranslational modification, and finally protein degradation (Balch et al., 2008; Powers et al., 2009). Any malfunction of proteostasis is expected to impair function by reducing activity, metabolic flow, and protein stability.

Since many regulatory factors have been shown to participate in the posttranslational control of GluTR, it is generally accepted that GluTR is the rate-limiting enzyme in ALA synthesis (McCormac et al., 2001). ALA synthesis capacity would be expected to correlate with the amount of the most complex and highly regulated enzyme in the pathway. Indeed, a previous study indicated that ALA synthesis is associated with soluble GluTR, while the remaining portion of GluTR is associated with the membrane (Schmied et al., 2018). The current analysis of GSAAT offers additional information on the control of ALA synthesis. Little was known about the contribution of GSAAT to the overall flow of ALA into the porphyrin-synthesizing branch of TBS. Interestingly, the ALA synthesis capacity was minimized in the absence of GSAAT2 under all four light regimes in SD and CL (Figures 2, G and 3, E), although GluTR accumulated to higher levels. However, the elevated GluTR content was not reflected in an increased capacity for ALA synthesis. The decreased ALA synthesis rate in *gsa* mutants due to lower in-planta GSAAT activity points to a significant role for GSAAT, particularly GSAAT2, in the overall capacity for ALA synthesis. In our study, the accumulation of GSAAT correlated strictly with the activity of GSAAT, with ALA synthesis, and with the corresponding chlorophyll contents in leaves of wild-type, *gsa1* and *gsa2*. We therefore conclude that besides GluTR, the amount GSAAT and additional regulatory mechanisms determine the rate of ALA synthesis.

Several factors are known to posttranslationally modulate GluTR activity and contribute to a distribution of GluTR in the stroma and membrane-associated fractions (see “Introduction”). The soluble portion of GluTR is considered to correlate with ALA synthesis, where GSAAT and additional regulatory factors mutually define/control ALA synthesis. Based on our results, we hypothesize that the levels of GSAAT also define the amount of GluTR, which is assembled in the ALA-synthesizing complex. As the decline in GSAAT content in *gsa1* and *gsa2* limits the ALA synthesis rate, GSAAT deficiency affects the quantitative contribution of GluTR to ALA synthesis. Strikingly, GSAAT2 deficiency modulates the GluTR content and the ALA synthesis rate in a divergent manner. We propose that excess GluTR in *gsa2* is disconnected from ALA synthesis and is sequestered at the membrane. Thus, GSAAT, mainly GSAAT2, exerts an influence on the level of active GluTR in the stromal fraction and hence the overall capacity for ALA synthesis.

The membrane-associated portion of GluTR (Schmied et al., 2018) is strongly protected against proteolysis (Apitz et al., 2016). As seen in the deregulated *GSA2* mutants, it is assumed that inactive GluTR is deposited on the membrane. The elevated GluTR level relative to wild-type could be explained by enhanced stability of the membrane-bound GluTR due to its interactions with other proteins, such as FLU, but the impact of other proteins cannot entirely be excluded. As a result, the membrane-associated GluTR is apparently less accessible to the soluble Clp protease and its selector proteins (Apitz et al., 2016). This was confirmed by the finding that the ratio of membrane bound to soluble GluTR increased in response to ALA supplementation and heme-dependent proteolysis in *gsa2* compared to the wild-type (Supplemental Figure S5). Apart from the higher accumulation of GluTR in GSAAT2-deficient mutants, a more pronounced change in the distribution of GluTR between the stroma and membrane fraction was detected. We propose that the level of GluTR required for ALA synthesis is reflected in the ratio of stromal to membrane-bound GluTR in light-exposed leaves.

While GSAAT2 deficiency in *gsa2* correlated with a lower ALA synthesis rate and a higher level of GluTR accumulation in the membrane fraction, overproduction of GSAAT1 in *gsa2* also led to increased GluTR accumulation. These observations point to modulated homeostasis for ALA synthesis. We proposed that stoichiometric amounts of both GluTR and GSAAT are required to enable the formation of a stable ALA-synthesizing protein complex. Balanced expression of *GSA1* and *GSA2* apparently ensures appropriate ALA synthesis in the wild-type, and in the event of a shortage of GSAAT2, the excess GluTR is stored on the membrane. In summary, we suggest that the amount of GSAAT controls the level of GluTR that contributes to ALA synthesis.

The ALA-induced degradation of GluTR in darkness observed by Richter et al. (2019) was confirmed in the current study (Supplemental Figure S4). Interestingly, supplementation of the *gsa2* mutant with ALA stabilized GluTR and GBP in the membrane fraction compared to wild-type seedlings. This finding supports the conclusion that the lack of stromal GSAAT2 has an important effect on the distribution of GluTR, increasing the membrane-bound fraction at the expense of GluTR in the stroma.

In contrast to the increase in GluTR proteolysis induced by ALA feeding, the provision of exogenous ALA for 24 h did not result in any significant degradation of GSAAT content in wild-type or the *gsa* mutants (Supplemental Figure S4), and its content in the stromal fraction remained similar to that of the *T* = 0 samples. Moreover, other representative TBS proteins were not degraded as a result of ALA feeding and the accumulation of additional free heme in plastids during dark incubation (Richter et al., 2019).

In conclusion, the GSAAT content correlates with GSAAT activity and the ALA synthesis rate, and consequently with the chlorophyll contents in *gsa* and wild-type seedlings. The total amount of GluTR reflects the amounts of FLU-mediated membrane-bound and soluble fractions. The latter is likely to be more accessible to proteolytic degradation. Therefore, the GSAAT content modulates the amount of soluble GluTR available for ALA synthesis.

### The GSAAT interaction in an ALA-synthesizing complex

The expression of *GSA* and *HEMA* genes is controlled in parallel during photoperiodic growth (Supplemental Figure S1, C and D), most likely to ensure the availability of equivalent amounts of both proteins for ALA synthesis. The physical interaction of GluTR and GSAAT was previously postulated to explain efficient substrate channeling (Moser et al., 2001), although experimental evidence for the direct interaction of both proteins in the control of ALA synthesis in plants was lacking. Our results explain why the interdependence between GluTR and GSAAT was more difficult to demonstrate in a stable complex for plant ALA synthesis. We demonstrated the interaction of GSAAT with GluTR and GBP by both pull-down and bimolecular fluorescence complementation (BiFC)-based assays (Figure 5, A and B). Moreover, besides the tight GluTR–GBP interaction, which was confirmed in an inverse pull-down experiment with GluTR as bait protein (Supplemental Figure S5; Czarnecki et al., 2011), GSAAT was also found in the eluate (Figure 5D). As GSAAT also interacts with GBP, we hypothesize that GBP forms the connecting and stabilizing link between GSAAT and GluTR.

Ultimately, our cross-linking experiment highlighted enhanced cross-linker–assisted interactions among the three proteins, while stable connections between dual combinations of the proteins were only detectable between GluTR and GBP. This latter interaction previously led to the identification of GBP (Czarnecki et al., 2011). As the cross-linking experiment did not establish any other bilateral connections among the three proteins, this may explain why previous attempts failed to show any GluTR–GSAAT interaction. Here, assays performed with total plant extracts or in vitro, together with recombinant GBP, were apparently more likely to provide sufficient stability to the ALA-synthesizing protein complex containing GluTR and GSAAT (Figure 5;Supplemental Figures S5 and S6). However, we cannot exclude the possibility that the bilateral interaction of GSAAT and GluTR in the pull-down experiments with plant extracts and upon their transient expression in the BIFC assays was stabilized by GBP. Thus, our findings suggest that GBP mediates the stable association of GluTR and GSAAT in a tripartite protein complex (Figure 6). Using native 2D BN-SDS–PAGE, substantial portions of the three proteins (GluTR, GSAAT, and GBP) were found to co-migrate in the same high-molecular-mass fraction of 540 kDa, which is the assumed molecular weight of the stromal complex involved in ALA synthesis.

Ultimately, the microscale thermophoresis (MST) experiments showed the strength of interactions among GBP, GluTR, and GSAAT. The dissociation constants deduced from the MST experiments reflect a decreasing affinity of the interactions in the analyzed protein complex in the order of GBP–GluTR, GBP–GSAAT, GBP/GluTR–GSAAT, and GluTR–GSAAT. The *K*_D_ values of GluTR and GBP confirm the previously defined tight affinity (Czarnecki et al., 2011) and suggest that GBP mediates the interaction between GluTR and GSAAT. This is consistent with the results of different pull-down experiments showing the intermediary role of GBP. Without the contribution of GBP, only a loose interaction of GluTR–GSAAT was detected (Supplemental Figure S7). It remains to be elaborated in future experiments how efficient ALA synthesis is achieved without GBP, as the allelic *gbp/pgr7* mutants showed very little reduction in ALA synthesis rates (Jung et al., 2010; Hou et al., 2019).

The proposed model (Figure 6), which is based on published structures of all three dimeric proteins (Protein Data Bank [PDB] (https://www.rcsb.org/) ids 4n7r and 5hdm) and the calculations derived from interfaces identified by cross-linked peptide motifs of GluTR, GSAAT, and GBP, consists of two GluTR, two GBP, and four GSAAT molecules (resulting in molecular masses of 106 kDa, 62 kDa, and 184 kDa, respectively, for a total of 352 kDa). Inside the open triangle of the Y-shaped GluTR dimer, a GBP dimer is placed. Its C-terminal part is cross-linked with GSAAT, which confirms a GBP–GSAAT interaction, likely at both flanks of the Y-shaped GluTR dimer. The scaffolding of GBP creates interfaces between GluTR and GSAAT, which would allow the direct exchange of metabolites.

The discovery of this protein complex closes a gap in our understanding of how adequate rates of ALA synthesis for chlorophyll and heme synthesis are achieved. In summary, we propose that the GSAAT–GluTR interaction is stabilized by the assistance of a scaffolding GBP. However, as GluTR, GSAAT, and GBP apparently co-migrate in a protein complex of 540 kDa, was cannot exclude the possibility that other auxiliary factors are needed to permit substrate channeling between GluTR and GSAAT in order to balance ALA synthesis.

## Materials and methods

### Plant materials and growth conditions

The *A. thaliana* T‐DNA insertion mutants *gsa1* (SAIL_1223B02) and *gsa2* (GABI_364C09) obtained from the Nottingham Arabidopsis Stock Centre were verified by genotyping using the primers listed in Supplemental Table S3. To construct the *35S:GSA1* gene, *GSA1* was amplified from Arabidopsis Col-0 genomic DNA, cloned into the entry vector pJet1.2 (Thermo Scientific), and then into pCAMBIA-Strep. The recombinant vector was transformed into *gsa2* using *Agrobacterium tumefaciens* strain GV2260 via the floral dip method. Arabidopsis seedlings were grown in soil under SD conditions (8-h light/16-h dark) and a standard light intensity (120 μmol photons m^−2^ s^−1^) or in CL at 10 (low light (LL)), 120 (standard light (SL)), and 300 (high light (HL)) µmol photons m^−2^ s^– 1^ in Conviron walk-in growth chambers (Winnipeg, Canada) using a combination of fluorescent tubes designated silhouette high output f54t5/841 ho from Philips and Grolux fho24w/t5/gro from Sylvania.

### Phylogenetic analysis

The GSA aminotransferase protein sequences from 13 different species, including four dicot and monocot plants, *C. reinhardtii*, *E. coli*, the moss *Physcomitrium patens*, and fission yeast (*Schizosaccharomyces pombe*) were used to construct the phylogenetic tree. Multiple sequence alignment of these GSA genes was carried out with the Muscle algorithm (Supplemental File S1). The phylogenetic tree was constructed using the Maximum Likelihood method and the Tamura–Nei model. The rooted tree with the highest log likelihood (−40949.82) and 100-bootstrap replications was used for phylogeny inferences. Evolutionary analyses were conducted in MEGA11 software, and the phylogenetic tree in Newick format is provided in Supplemental File S2.

### Nucleic acid extraction

Arabidopsis leaf tissues (20 mg) were homogenized in 200-μL extraction buffer (200-mM Tris pH 8.0, 150-mM NaCl, 25-mM EDTA, and 0.5% SDS), followed by centrifugation at 13,053 rpm, 4°C for 4 min. The supernatant was transferred to a new tube, an equal volume of isopropanol was added, and the mixture was centrifuged at 13,053 rpm and 4°C for 15 min. The precipitated DNA was washed twice with 70% ethanol and dissolved in 30-μL ddH_2_O. RNA was extracted using citric-acid buffer as described previously (Onate-Sanchez and Vicente-Carbajosa, 2008).

### qRT–PCR analysis

Complimentary DNAs were synthesized from 2-μg aliquots of RNA pretreated with DNase I (Thermo Scientific), using oligo(dT) primers and RevertAid reverse transcriptase (Thermo Scientific). qRT–PCR was performed using SYBR Green PCR master mix (Biotool) under the cycling conditions (initial denaturation at 95°C for 30 s [denaturation at 95°C for 10 s; annealing and elongation 60°C for 30 s, 40 cycles]; denaturation at 95°C for 30 s; melting curve at 60–95°C for 5 s/0.5°C after each run) on a CFX96 real‐time system (Bio‐Rad). Expression levels were calculated by the 2^△△Ct^ method and normalized to the reference gene *SAND*. qRT–PCR primers used in this study are listed in Supplemental Table S3.

### Protein extraction and immunoblot analysis

Four‐week‐old Arabidopsis leaf tissue (20–30 mg) was frozen in liquid nitrogen and homogenized. The samples were dissolved in 200–300 μl 2 × SDS–PAGE sample buffer (100-mM Tris/HCl pH 6.8, 4% SDS, 20% glycerol, and 2-mM dithiothreitol) at 95°C for 10 min and centrifuged for 1 min at 13,053 rpm at room temperature. Aliquots (10–15 μL) of each sample were then loaded onto an SDS–PAGE for subsequent immunoblot analysis. The antibodies were either purchased (Thermo Fischer [GST-tag] and Sigma [His-tag]) or produced from purified antigens (GSAAT, GluTR, GBP, FLU, ALAD, coproporphyrinogen oxidase [CPOX], PPOX, FC2, FC1, genomes uncoupled 4 [GUN4], and PORB) generated in our laboratory.

### ALA feeding experiments and fractionation of soluble and membrane-bound Arabidopsis proteins

Arabidopsis leaves (20 mg) were incubated in 20-mM TRIS buffer (pH 7.4) with or without 1-mM ALA in the dark for 24 h, dried on a paper towel, and frozen in liquid nitrogen for subsequent analysis.

Homogenized leaf material was resuspended in 200 µL of phosphate-buffered saline buffer (PBS; 20-mM sodium phosphate, 150 mM NaCl, pH 7.4), and 100 μL of the suspension was immediately frozen in liquid nitrogen for subsequent analysis of protein content. The rest of the sample was incubated on ice for 10 min to ensure complete lysis of chloroplasts. Stromal and membrane fractions were separated by centrifugation at 13,053 rpm and 4°C for 10 min. The supernatant containing soluble stromal proteins was transferred to a new tube and mixed with 100 μL 2 × SDS–PAGE sample buffer. The pellet comprising membrane-associated proteins was resuspended in 500 μL of PBS, incubated on ice for 10 min, centrifuged again, and dissolved in 100 μL of 2 × Laemmli buffer and 100 μL PBS. Samples of total proteins were mixed with 100 μL of 2 × Laemmli buffer.

### Determination of heme and chlorophyll contents

Chlorophyll was extracted from frozen leaf tissue using ice-cold alkaline acetone (acetone:0.2 N NH4OH, 9:1) at 4°C, and centrifuged (12,210 rpm, 20 min, 4°C). The noncovalently bound heme was extracted from the pellet with acetone: 37% HCl: dimethylsulfoxide (AHD, 100:20:5) buffer at room temperature and centrifuged, as described previously (Czarnecki et al., 2011). Both supernatants (for chlorophyll and heme) were centrifuged a second time at 12,210 rpm for 15 min, then analyzed and quantified by high‐performance liquid chromatography (HPLC) using the Agilent 1100 or 1290 HPLC system equipped with a diode array and fluorescence detectors (Agilent Technologies), essentially as described (Wang et al., 2021). The Agilent ChemStation for liquid chromatography system (product no. G2170BA) was used in the HPLC analyses.

### Chloroplast isolation

Twenty gram Arabidopsis leaves were homogenized in a Waring blender with 200 ml of chloroplast homogenization buffer (0.45-M sorbitol, 20-mM Tricin-KOH pH8.4, 10-mM EDTA, 10-mM NaHCO_3_ and 0.1% BSA) at 4°C. The total extracts were filtered through two layers of Miracloth, and the chloroplasts were pelleted by centrifugation at 4°C, 500*g* for 20 min. The supernatant was completely discarded, and the pellet was re-suspended in 500-μL chloroplast washing buffer (CWB) containing 0.3-M sorbitol, 20-mM Tricine-KOH, 2.5-mM EDTA, and 5-mM MgCl_2_ by gently shaking the centrifuge tube. The chloroplasts were then loaded onto a gradient of 14 mL 40% Percoll and 5 mL 80% Percoll and centrifuged at 4°C, 6,581 rpm for 30 min in a swing-out rotor. After centrifugation, the intact chloroplasts in the interlayer of the gradient were removed with a truncated 1-mL tip and transferred to 20-mL CWB buffer. Finally, chloroplasts were collected by centrifugation at 4°C, 2,307 rpm for 5 min and stored at −80°C.

### Analysis of photosynthetic complexes by 2D BN–PAGE

The photosynthetic complexes were obtained from total chloroplast extracts instead of thylakoids by solubilization with 1% n-dodecyl-β-d-maltoside (DM) and subsequently analyzed by BN**–**PAGE as described earlier (Järvi et al., 2011).

### Assay of GSAAT activity

GSAAT assays were performed as described previously (Hoober et al., 1988) with some modifications. The crude extracts for the enzyme assay were prepared by homogenizing the tissue in 0.1-M MOPS buffer (Na 2-(N-morpholino)ethanesulfonate-0.1 M Na phosphate) pH 6.8. Aliquots of the extract (200–300 µg) were combined with 10–30 µM GSA, 10 µM PLP, and 10-mM levulinic acid in a total volume of 1 mL and incubated at 28°C for 10 min. The reaction was terminated by the addition of ethyl acetoacetate and adjusted to pH 6.8, followed by heating for 10 min at 100°C. The tubes were then cooled to room temperature and 1 volume of modified Ehrlich’s reagent (12.6% perchloric acid, 74.6% acetic acid, 11.4% HgCl_2_, and 0.4% 4-NN-dimethylamino)benzaldehyde) was added and finally, absorption was recorded at 553 and 526 nm as described by Mauzerall and Granick (1956).

### Quantification of ALA synthesis

Aliquots (30–40 mg) of Arabidopsis leaf material were incubated under SD conditions in 50-mM Tris–HCl buffer (pH 7.2) containing 40-mM levulinic acid (pH 7.5) for 3–4 h and subsequently homogenized in 450 μL of 20-mM K-phosphate buffer (pH 6.8). After centrifugation, 400 μL of the supernatant was mixed with 100 mL of ethyl acetoacetate and boiled for 10 min. Finally, 500 mL of Ehrlich’s reagent was added, and ALA derivatives were quantified at λ 553 nm (Mauzerall and Granick, 1956).

### Bimolecular fluorescence complementation assay

Full-length cDNA copies of target genes were cloned into the pJET2.1 vector (Thermo Scientific) using gene-specific primers (Supplemental Table S3) and later transferred into the pVyNE and pVyCE plasmids (Gehl et al., 2009; Invitrogen, Carlsbad, CA, USA), thus fusing them to either the N-terminal or the C-terminal half of YFP-Venus protein. For BiFC experiments, the constructs were transformed into *A. tumefaciens* GV2260 and the tagged interaction partners were transiently co-expressed following infiltration into *N. benthamiana* leaves. Finally, after expressing the proteins for 48–72 h in the dark, at least three leaf discs from infiltrated tobacco plants were analyzed for the YFP signals under an LSM 800 confocal microscope (Zeiss; λex 514 nm, λem (YFP) 530–555 nm, λem (Chl) 600–700 nm).

### Expression and purification of recombinant proteins

GST-GluTR, His-GSAAT and GST, His-GBP were expressed and purified from *E. coli* cultures (strain Rosetta 2) strain as described previously (Czarnecki et al., 2011; Apitz et al., 2016; Song et al., 2016; Wang et al., 2018) using Ni-NTA (Thermo Scientific) and Glutathione Sepharose 4B (Cytiva) resins for HIS and GST-tag purification, respectively. All purification steps were performed according to the manufacturer’s protocol. The proteins were dialyzed and concentrated in PBS buffer using Amicon Ultra-4 30K centrifugal filter devices and stored at −80°C for further use.

### Pull-down experiments

Pull-down assays were performed as described by Wang et al. (2018), with some modifications. In vivo GST-pull-down assays were performed by incubating 50 µg of purified recombinant GST–GluTR with 1% n-DM solubilized plastid lysates (100 μg chlorophyll) in binding buffer (BF: 25 mM Tris–HCl, pH 7.8, 150-mM NaCl, 5-mM MgCl_2_, 10% (v/v) glycerol and cOmplete protease inhibitor cocktail, MedChem Express) overnight at 4°C at 100 rpm. After adding 50 µL glutathione-Sepharose 4B (Cytiva), the sample was incubated for 1 h at 4°C at 100 rpm. The resins were separated from the plastid lysates by centrifugation at 3,000 rpm for 5 min at 4°C, and washed five times with BF. Finally, the proteins bound to the GST-Sepharose were eluted with BF supplemented with 10 mM reduced glutathione and then separated by SDS–PAGE and analyzed by immunoblotting with the indicated antibodies. The in vitro GST pull-down assays were performed by incubating recombinant GST–GluTR, GST–GBP, and GST proteins with purified 6xHis-GSAAT and 6xHis-GBP proteins in BF overnight at 4°C at 100 rpm. Other pull-down assays were carried out by incubating the plastid lysates (100-μg chlorophyll), which were previously DM-solubilized in BF, with 50 µg His-GSAAT recombinant proteins. After adding 50 µL of Ni-NTA agarose (Thermo Fisher Scientific) to all tubes, the samples were incubated for 1 h at 4°C at 100 rpm. The resins were washed as described previously with BF containing 20-mM imidazole. Finally, the bound proteins were eluted in binding buffer supplemented with 200 mM imidazole, followed by 10% SDS–PAGE and immunoblotting analysis.

### Cross-linking experiments

GluTR, GBP, and GSAAT were mixed either pairwise or together and incubated with disuccinimidyl sulfoxide (DSSO, 1 mM in DMSO, Thermo Scientific) in PBS, or in a mock solution, for 1 h at room temperature in PBS. Excess cross-linker was quenched by the addition of Tris (20 mM fc). For mass spectrometry (MS) analysis, proteins were prepared following a modified filter-aided sample preparation protocol, reduced with Tris(2-carboxyethyl)phosphine, and alkylated with 2-chloroacetamide. The proteins were then digested with trypsin (1:50) overnight at 37°C. Liquid chromatography–MS/MS analysis was performed on an EASY-nLC 1200 (Thermo Fisher) coupled to a Q Exactive HF mass spectrometer (Thermo Fisher). Separation of peptides was performed on 25-cm frit-less silica emitters (New Objective, 0.75-µm inner diameter), packed in-house with reversed-phase ReproSil-Pur C18 AQ 1.9-µm resin (Dr. Maisch). The column was kept at a constant temperature of 50°C. Peptides were eluted over a period of 78 min, applying a segmented linear gradient of 0%–98% solvent B (solvent A 0% ACN, 0.1% FA; solvent B 80% ACN, 0.1% FA) at a flow rate of 300 nL min^−1^. Mass spectra were acquired in data-dependent acquisition mode. For fragmentation, only precursors with charge states 2–6 were considered, and up to 20 dependent scans were taken. For dynamic exclusion, the exclusion duration was set to 40 s and a mass tolerance of +/− 10 ppm was set. The isolation window was set to 1.6 *m/z* with no offset. A normalized collision energy of 30 was used. MS2 scans were taken at an Orbitrap Resolution of 15,000, with a fixed First Mass (*m/z*) = 120. Maximum injection time was 22 ms and the normalized AGC Target 50%. Raw files were searched using MaxQuant (MQ) v2.0.3.0 against the sequences of the three proteins of interest as they were expressed in bacteria. Cross-links between Lys, Ser, Thr, and Trp were allowed for DSSO as the MS cleavable linker. Peak refinement was activated, cross-link FDR was set to 0.05, four missed cleavages were allowed, and a maximum peptide mass of 6,000 Da was allowed. Default software settings were used otherwise. For docking via the Haddock 2.4. webserver, 3D structures for the protein constructs were predicted using AlphaFold 2.1.1 via Colab. Overhangs with a pLDDT <70 were trimmed prior to uploading the PDB files. Two copies of each protein were used in the docking to resemble previously published crystal structures, and cross-links identified via MS by MQ were used as ambiguous restraints between all possible monomers. The crystal structure of the GluTR–GBP complex with PDB identifier 5YJL was superimposed with a docking result based on GBP with Pymol. In the second step, the GSAAT structures from the docking were superimposed upon GSAAT dimers from PDB structure 5HDM. Cofactors were derived from the respective crystal structures, and active-site residues of GluTR (Cys 144) and the Schiff-base-reactive Lys 274 of GSAAT (Song et al., 2016) are displayed as red spheres.

### Microscale thermophoresis experiments

6xHis-GBP and 6xHis-GSAAT was labeled using the Protein Labeling Kit RED-NHS 2nd Generation (NanoTemper; Munich, Germany) according to the manufacturer’s instructions. Briefly, 20 µM of 6× His-GBP recombinant proteins were incubated with NHS labeling buffer and 120 µM of the dye RED-NHS second generation at room temperature for 30 min in dark. The labeled proteins were eluted by loading them onto an equilibrated B column resin by adding 500 µL of PBS buffer. The degree of labeling of the proteins was checked using UV–Vis spectrophotometry at 650 and 280 nm and was approximately 0.5–0.7. Ten to 20 µM of ligand 6xHis-GSAAT were mixed with the labeled target proteins 6×His-GBP with or without GluTR in a series of 1:1 dilutions, producing ligand concentrations ranging from 5 nM to 110 μM. After 5 min of incubation, the samples were loaded into Monolith NT.115 capillaries, and the thermophoresis was recorded in a NT.115 instrument (NanoTemper) at a constant temperature of 30°C, 40% light-emitting diode power and 100% excitation. MST signal from data of three different independent experiments was analyzed using MO.Affinity Analysis software v.2.1.3.

### Statistical analysis

The statistical analysis performed was a result of samples harvested and processed independently from at least three replicates from each genotype and/or the treatment under different light conditions. The data plotted as bar graphs with error bars were the mean of three replicates (*n* = 3) and the standard deviation calculated, respectively. Statistical significance compared between Col-0 and other genotypes (*gsa1, gsa2, 35S:GSA1*) at CL-M light condition and CL-L or CL-H is indicated by ^a^*P*≤ 0.05, ^b^*P* ≤ 0.01, ^c^*P* ≤ 0.001 obtained using Student’s *t* test.

### Accession numbers

Sequence data from the article can be found in the GenBank/EMBL libraries under the following accession numbers: *AtGSA1* (AT5G63570) and *AtGSA2* (AT3G48730). The raw data are available on ProteomeXchange and jPOST under the identifier JPST001362. For review purposes, these credentials can be used to access the files: URL https://repository.jpostdb.org/preview/6106523276193d2f1b13dd, Access key: 4843

## Supplemental data

The following materials are available in the online version of this article:

**Supplemental Figure S1.** Phylogenetic tree of GSAAT and expression profiles of the two GSA genes.

**Supplemental Figure S2.** Analysis of the transcript levels and protein contents of *GSA1, GSA2* and *HEMA1.*

**Supplemental Figure S3.** Analysis of the post-translational stabilization of proteins involved in ALA biosynthesis upon feeding with ALA.

**Supplemental Figure S4.** Photosynthetic protein complexes in the thylakoid membrane.

**Supplemental Figure S5.** Verification of protein-protein interactions of the proteins involved in ALA synthesis.

**Supplemental Figure S6.** In vitro pull-down experiments with purified recombinant GST-GluTR, GST-GBP, 6xHis-GBP and 6xHis-GSAAT proteins.

**Supplemental Figure S7.** Microscale thermophoresis (MST) experiments using 6xHis-GBP as target proteins.

**Supplemental Table S1.** Cross-linked peptides.

**Supplemental Table S2.** Dissociation constant values from the MST experiments.

**Supplemental Table S3.** Primers used in this study.

**Supplemental File S1.** Multiple Sequence Alignment of GSA genes in different species in Fasta format.

**Supplemental File S2.** Phylogenetic tree provided in Newick format.

**Supplemental Data Set S1.** Statistical data.

## Supplementary Material

koac237_Supplementary_Data

## Notes

B.G. and N.S. designed the experiments; N.S. performed most of the experiments. J.E. and I.F. performed the MS cross-linking experiments. N.S., J.E., I.F., and B.G. analyzed the data. B.G. wrote the manuscript with contributions from all authors. N.S., J.E., and I. F. gave critical comments and revisions to the manuscripts, all authors contributed to the proof reading. B.G. acquired funding for the research.

The author responsible for distribution of materials integral to the findings presented in this article in accordance with the policy described in the Instructions for Authors (https://academic.oup.com/plcell) is: Bernhard Grimm (Bernhard.grimm@rz.hu-berlin.de)
