# Supplementary material for: Glutamate 1-semialdehyde aminotransferase is connected to GluTR by GluTR-binding protein and contributes to the rate-limiting step of 5-aminolevulinic acid synthesis
Source: Plant Cell. 2022 Aug 16;34(11):4623–40. doi: 10.1093/plcell/koac237 (PMC9614494; doi:10.1093/plcell/koac237)
Supplement: koac237_Supplementary_Data [file koac237_supplementary_data.zip › koac237_Supplementary_Data/Supplemental Figures_24.07.pdf]

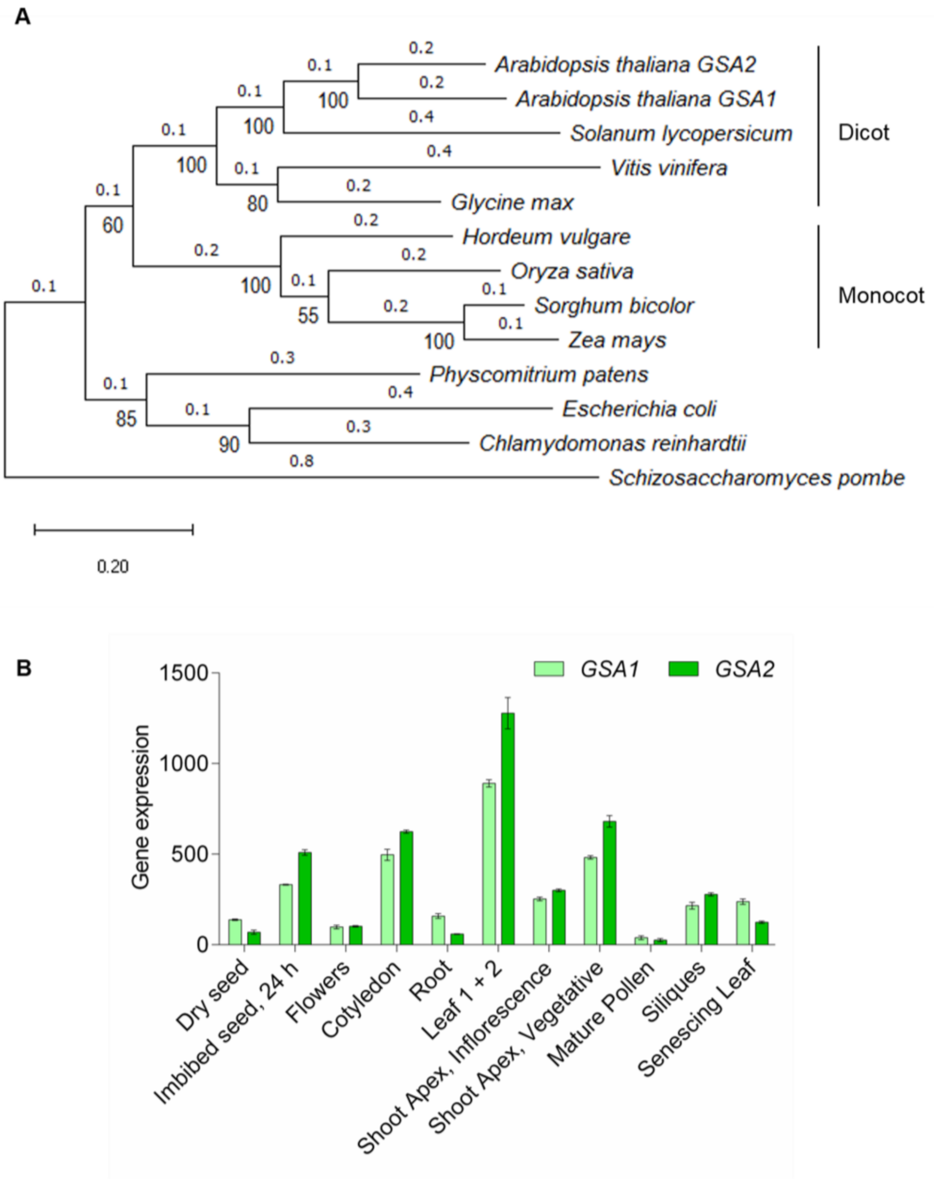

Supplemental Figure S1. Phylogenetic tree of GSAAT and expression profiles of the two *GSA* genes. (Supports Figure 2.) A Phylogenetic tree of the *GSA* homologs identified by BLAST. Based on a multiple sequence alignment created by MUSCLE (Edgar et al., 2004; Supplemental File S1). The phylogenetic tree was generated with the Maximum Likelihood Method and Tamura-Nei based model. This analysis involved 13 protein sequences from *Chlamydomonas reinhardtii*, *Escherichia coli*, fission yeast (*Schizosaccharomyces pombe*) and several photosynthetic organisms, i.a. moss (*Physcomitrium patens*) and several monocots and dicots. The tree was drawn to scale, with branch lengths measured in the number of substitutions per site (above the branches). The percentage of trees in which the associated taxa clustered together was shown below the branches. The phylogenetic tree was visualized with the help of the MEGA-11 program (Tamura et al., 2021) and a Newick machine-readable tree file was provided as a Supplemental File S2. **B** Expression profile of the two GSA genes in Arabidopsis. Publicly available data from Arabidopsis eFP Browser (http://bar.utoronto.ca/efp2/Arabidopsis/Arabidopsis_eFPBrowser2.html) which is based on a high-resolution RNA-sequencing profile of *Arabidopsis thaliana*, were used to depict the relative expression levels of the two GSA genes in different organs and at different developmental stages.

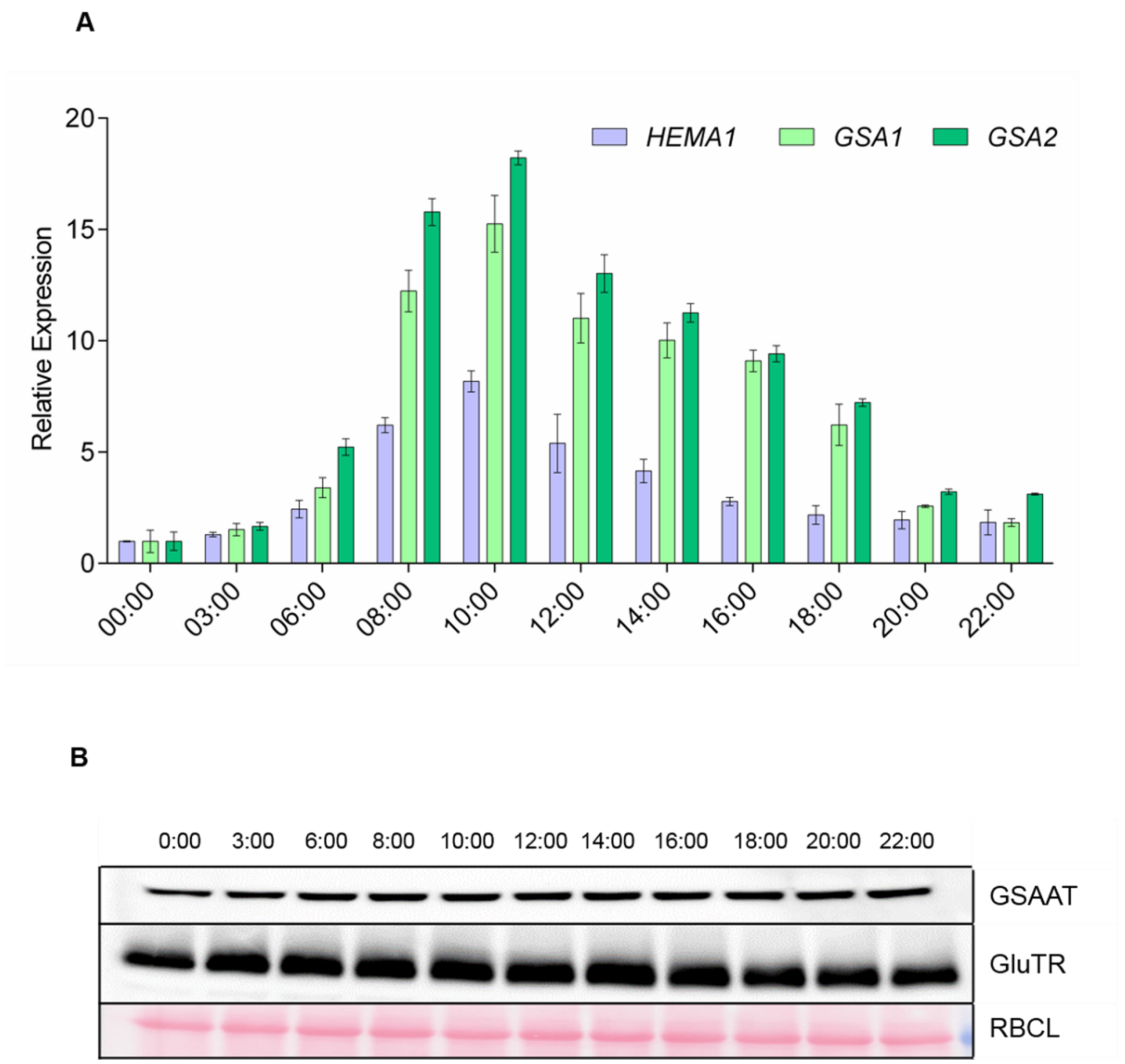

Supplemental Figure S2. Analysis of the transcript levels and protein contents of *GSA1*, *GSA2* and *HEMA1*. (Supports Figure 2.) A,B Analysis of the transcript levels (**A** qRT-PCR) and corresponding protein contents (**B** immune analysis) of the genes *GSA1, GSA2* and *HEMA1* in three-week-old wild-type seedlings grown under standard light intensity (120 µmoles photo m^{-2} s^{-1}) in short-day conditions. **A** Relative transcript levels are normalized to 1 at the time 0:00 (8 h before the onset of light) and calculated relative to *SAND* as the house-keeping reference gene. The data are plotted as mean +/- s.d. (n=3 independent biological repeats). **B** Immunoblot analysis of GluTR1 and GSAAT in wild-type (Col-0) over a 24-h period under SD conditions and standard light intensity (120 µmoles photon m^{2-} s^{-1}). Lanes with different time points always represent identical amounts of leaf material based on fresh weight as seen by RBCL: Ponceau S stain of the large subunit of RuBisCo, *HEMA1* encodes GluTR1, glutamyl-tRNA reductase; *GSA1* and *GSA2* encode glutamate-1-semialdehyde aminotransferase 1 and 2, RBCL, large subunit of the ribulose 1,5-bisphosphate carboxylase/oxygenase (RuBisCO), serves a loading control.

Supplemental Data. Sinha et al. (2022).Glutamate 1-semialdehyde aminotransferase is connected to GluTR by GluTR-binding protein and contributes to the rate-limiting step of 5-aminolevulinic acid synthesis. Plant Cell.

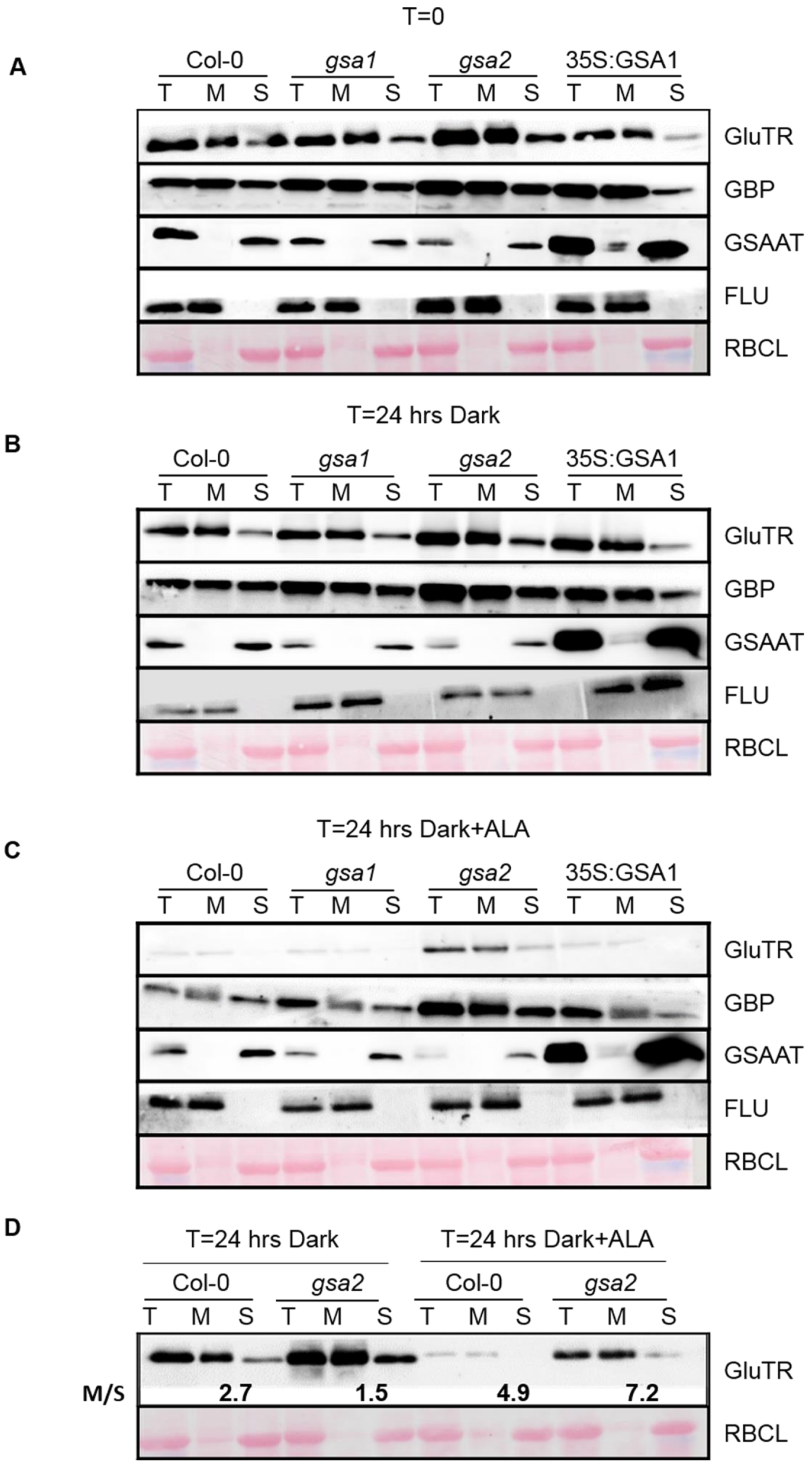

Supplemental Figure S3. Analysis of the post-translational stabilization of proteins involved in ALA biosynthesis upon feeding with 5-aminolevulinic acid. (Supports Figure 2.) A-D Immunoblot analysis of the stability and distribution of proteins involved in ALA biosynthesis in wild-type (Col-0), *gsa1, gsa2* and 35S:GSA1 lines incubated with and without 5-aminolevulinic acid (ALA) in the dark. Three-week-old, light-grown Arabidopsis seedlings of all four genotypes were incubated in buffer with (+) or without 1 mM ALA (+) in darkness. Samples were harvested prior to treatment (**A** T=0) and after 24 h in the absence (**B** T=24 h dark) or presence (**C** T=24 h + ALA). Immunoblot analysis of proteins involved in ALA biosynthesis of TBS (FLU, GluTR, GBP, GSAAT). **D** Quantitative immune analysis of GluTR in leaves of wild type (Col-0) and *gsa2* seedlings, which were incubated in darkness in either mock solution (T=24 hrs dark) or in 1mM ALA (T=24 hrs dark + ALA). Proteins of total (T) plastid extracts as well in the membrane (M) and stroma (S) fraction were applied to SDS-PAGE. The changes in the distribution of GluTR between membrane (M) and stroma (S) fractions were analysed. M/S depicts the membrane to soluble ratio calculated by Image J software. T, S and M represent identical amounts of leaf material based on fresh weight visualized by RBCL: PonceauS stain of the large subunit of RuBisCo.

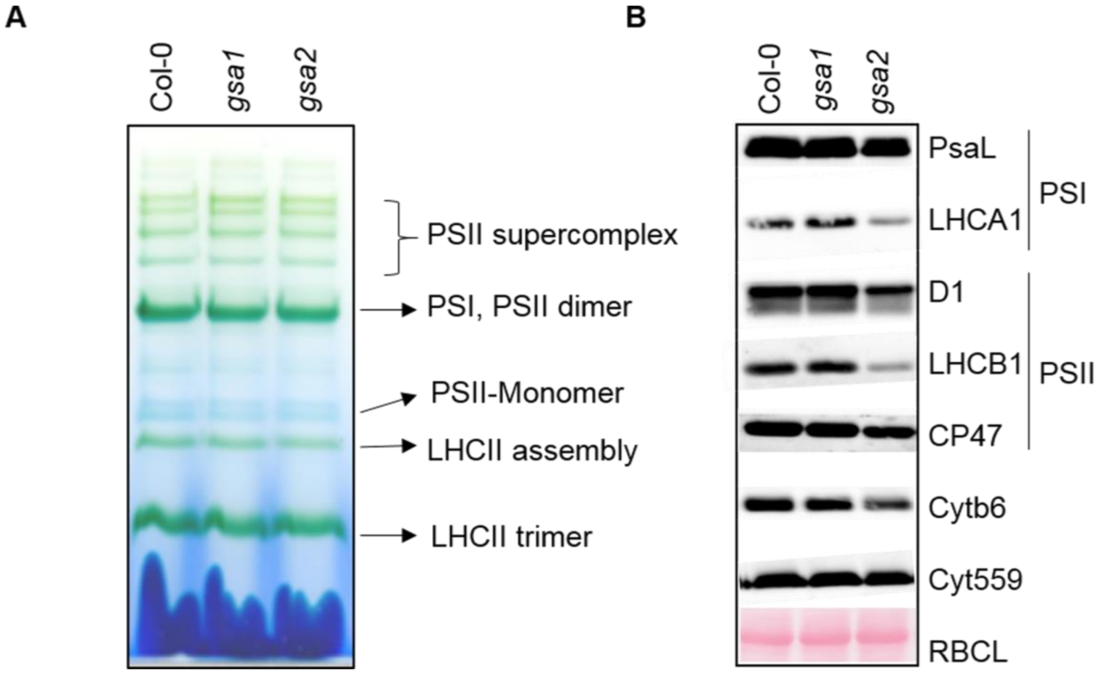

Supplemental Figure S4. Photosynthetic protein complexes in the thylakoid membrane. (Supports Figure 2.) A Chloroplast extracts from three-week-old seedlings of Col-0, *gsa1* and *gsa2* lines grown under SD and standard lighting conditions (120 µmoles photon m^{2-} s^{-1}) were fractionated on a 4-12% Blue Native- SDS-PAGE gel. The amounts of protein loaded onto the gel were normalized with respect to the chlorophyll level. The photosynthetic complexes are listed to the right of the respective band. **B** Immunological detection of different subunits of the photosynthetic protein complexes in total protein extracts after separation on 12% urea gels. Equal amounts of protein were applied to the gel. The Ponceau-stained large subunit of RuBisCO (RBCL) served as a loading control.

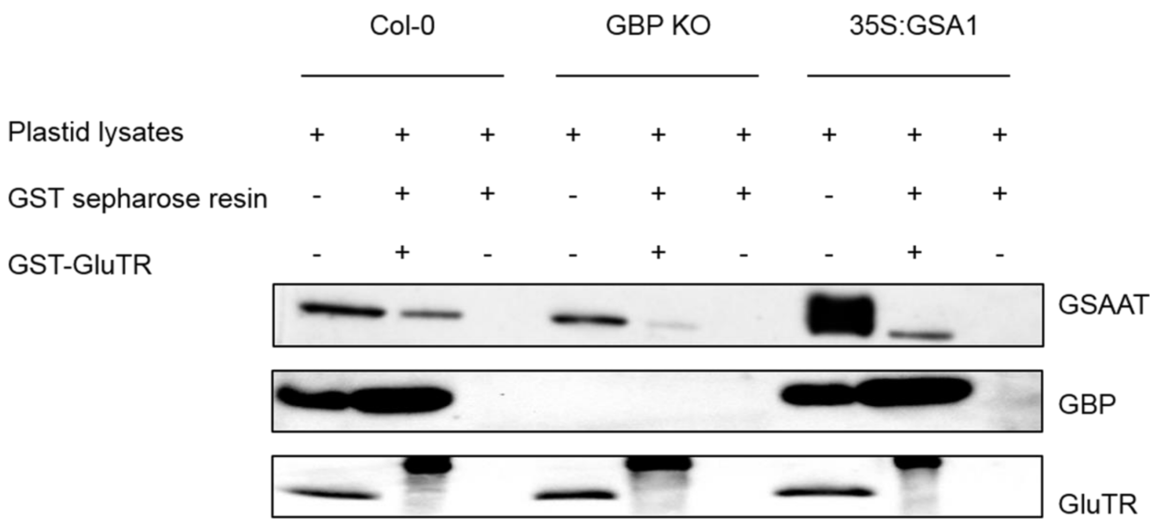

Supplemental Figure S5. Verification of protein-protein interactions of the proteins involved in ALA synthesis. (Supports Figure 5.) Analysis of in vivo pull-down experiments to demonstrate the differential interaction of recombinant purified GST-GluTR with GSAAT and GBP of the plastid lysates of wild-type (Col-0), *gbp* (GBP KO) and 35S:*GSA1*(*gsa2*) seedlings. The bait GST-GluTR was incubated with the protein extracts of these three Arabidopsis lines and glutathione-Sepharose 4B (Cytiva) resin. Finally, the proteins that are bound to GST-GluTR were subsequently eluted with buffer containing 10 mM reduced glutathione. The input and elution fractions were analysed on immunoblots using the indicated antibodies.

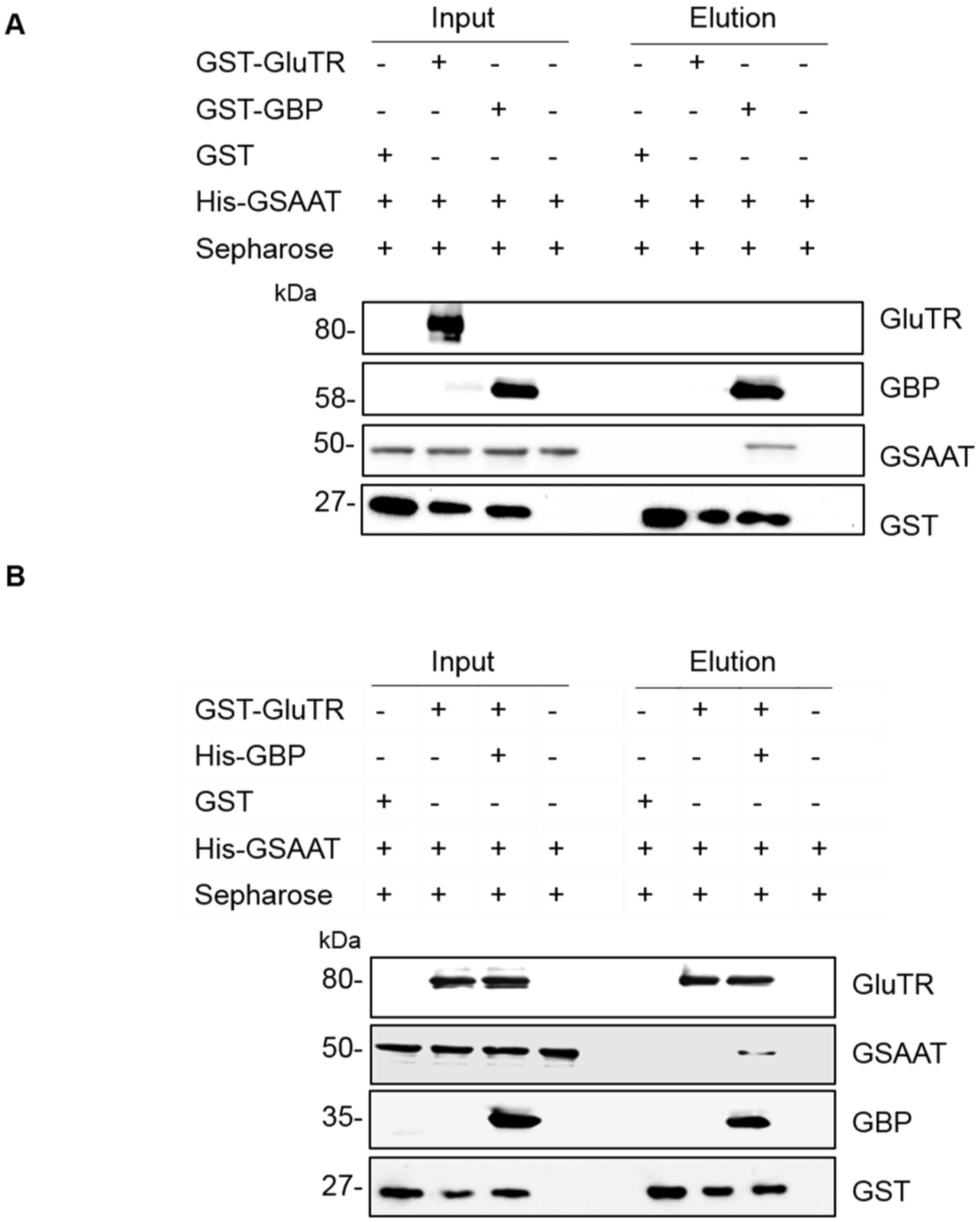

Supplemental Figure S6. In vitro pull-down experiments with purified recombinant GST-GluTR, GST-GBP, 6xHis-GBP and 6xHis-GSAAT proteins. (Supports Figure 5.) A and **B** In vitro, pull-down experiments with the recombinant bait protein GST-GluTR, GST-GBP, and GST on glutathione-Sepharose 4B (Cytiva) resin **A** and with GST-GluTR and as control with GST **B** which were incubated with purified recombinant prey protein 6xHis-GSAAT only **A**, and with His-GSA or 6xHis-GSAAT combined with 6xHis-GBP. After washing and elution with buffer containing 10 mM reduced glutathione, eluted proteins were analyzed after SDS polyacrylamide gel electrophoresis to demonstrate the differential interaction capacity of the recombinant bait proteins. An aliquot of the input and the eluted proteins immune-reacts with the antibodies raised against GluTR, GSAAT, GBP or GST, respectively.

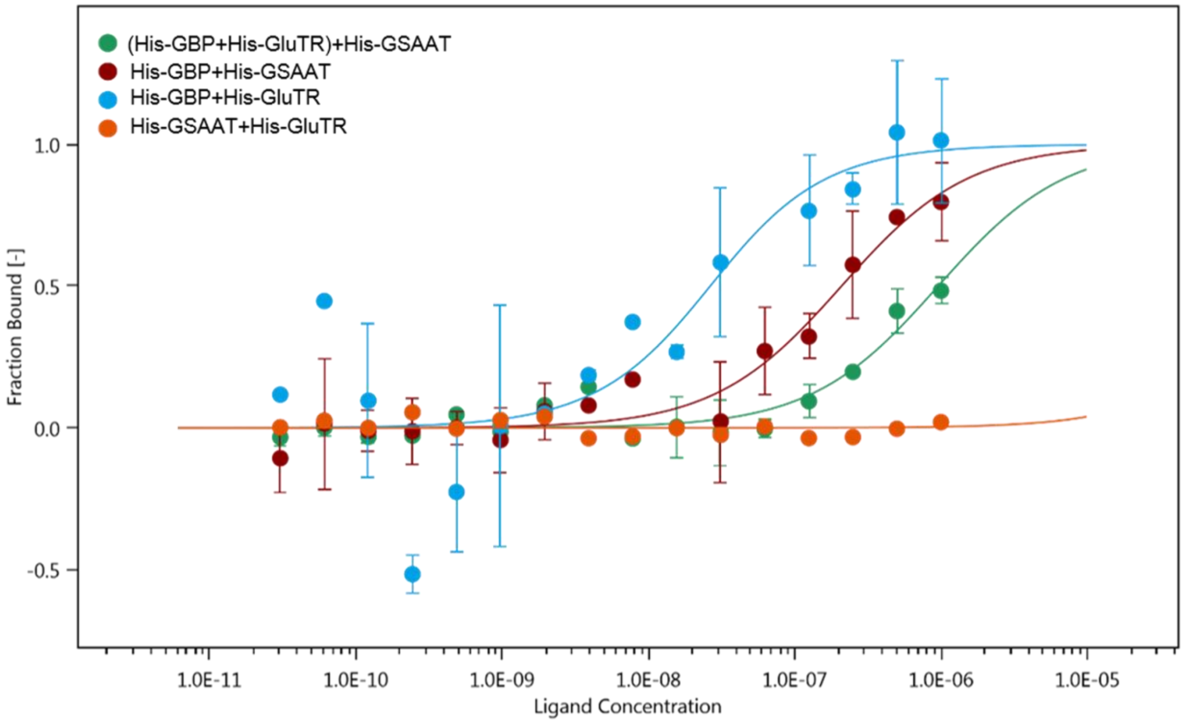

Supplemental Figure S7. Microscale thermophoresis (MST) experiments using 6xHis-GBP as target proteins. (Supports Figure 5.) Microscale thermophoresis (MST) of the fluorophore-labelled 6xHis-GBP with or without 6XHis-GluTR titrated against increasing amounts of 6xHis-GSAAT. To check the interaction between GSAAT and GluTR, 6xHis-GSAAT proteins were labelled and titrated against 6xHis-GluTR proteins. Due to the previous evidence of GBP-GluTR interaction (Czarnecki et al., 2011), the labelled 6XHis-GBP incubated with 6XHis GluTR was used as a positive control. Data are given as means of the standard deviation of three different independent experiments

Protein 1	Peptide Sequence 1	Peptide Position 1	Protein 2	Peptide Sequence 2	Peptide Position 2	Crosslink score
6XHis GSAAT	GSHMoxASMTGGQQMGR	19	6XHis GBP	SVSPDKR	85	11.114
6XHis GluTR	EKLAIPEAEWPR	62	6XHis GBP	IPFPMEVTDEKGAK	243	13.886
6XHis GluTR	MEIYVLALSQHR	98	6XHis GBP	EDSLYVVAVDR	137	9.261
6XHis GluTR	EVTEWMoxSK	116	6XHis GBP	GVYDVR	229	6.944
6XHis GluTR	VYNVDDLKEVVAANK	353	6XHis GBP	IPFPMEVTDEKGAK	243	26.641
6XHis GluTR	EVVAANKEDR	360	6XHis GBP	FAVDKDGTPVLCcmLNR	69	25.715
6XHis GluTR	EVVAANKEDR	360	6XHis GBP	IPFPMEVTDEKGAK	243	23.979
6XHis GluTR	EVVAANKEDR	360	6XHis GBP	TPQCcmTIQGSIGRPGDDTVLKR	119	21.993
6XHis GSAAT	ELTNGILEAGK	378	6XHis GBP	EDSLYVVAVDR	139	7.109
6XHis GluTR	AMAEQQQK	491	6XHis GBP	SVSPDKR	85	NA

Supplemental Table S1. Crosslinked peptides. **(Supports Figure 5.)** The identified list of crosslinked peptides, their positions and the crosslink score in different proteins used i.e. 6xHis-GSAAT, 6xHis-GBP and 6xHis-GluTR by LC-MS analysis.

Target	Ligand	K_D value
6XHis GBP	6XHis GluTR	42 nM
6XHis GBP+ 6XHis GluTR	6XHis GSAAT	940 nM
6XHis GBP	6XHis GSAAT	178 nM
6XHis GSAAT	6XHis GluTR	3 μM

Supplemental Table S2. Dissociation constant values from the MST experiments. (Supports Figure 5.) The dissociation constant (K_D) values between different target and ligand proteins were deduced by microscale thermophoresis experiments.

Primer Name	Primer Sequence (5'-3')	Usage
GSA1-Fw	AATCTAATGCCTGGAGGTGTG	Genotyping
GSA1-Rv	TAGCATGACCGTGATAACACC	Genotyping
GSA2-Fw	ACTTACGAGCGCAAGTGAGAG	Genotyping
GSA2-Rv	CACCACTGGTTGTCCACCAAC	Genotyping
35S:GSA1-Fw	CCCGGGACTTGACTTTCGTATTCGGGT	Complimentation
35S:GSA1-Rv	CCCGGGTAATCGGAAAGGCAAATCGGT	Complimentation
SAND-Fw	AACTCTATGCAGCATTTGATCCACT	qPCR
SAND-Rv	TGATTGCATATCTTTATCGCCATC	qPCR
GSA1-Fw	TCAAAGAAGAGCGACACAGAG	qPCR
GSA1-Rv	GTAAACACCTTCTTCCAACATTCC	qPCR
GSA2-Fw	GAATCACACCTGACTTAACAACTC	qPCR
GSA2-Rv	GATGTCTCTTCTTCCACCGT	qPCR
HEMA1-Fw	TTGCTGCCAACAAAGAAGAC	qPCR
HEMA1-Rv	CCGTCTCCAATGAATCCCTC	qPCR
GBP-Fw	ATCTAGACTTTGTGGTTTCAGAAA	qPCR
GBP-Rv	TGGAAATGGAATCCTCACATC	qPCR
FLU-Fw	AAGCCATACAGTATCACTCCA	qPCR
FLU-Rv	TCCAGAATCTTCACTTTCCCT	qPCR
FC2-Fw	CCATACGTTGGTGCTATGGCT	qPCR
FC2-Rv	CGAGCGGAACTAACGACTGTC	qPCR
GUN4-Fw	TTAGGACACTTACCGCTCAC	qPCR
GUN4-Rv	GCTCGTCTTCTGTTTCTCCA	qPCR
ALAD-Fw	CGATGAAGAGAAAGTGATGATGGA	qPCR
ALAD-Rv	CTATTCTACCGCTTCTCGCC	qPCR
PORB-Fw	TGATTACCCTTCAAAGCGTCTCA	qPCR
PORB-Rv	CAATGTATTCGTGTTCCCGGT	qPCR
PPOX I-Fw	TACTAGTACAATGTCGGCGACGCTTAC	BIFC
PPOX I-Rv	TCTCGAGCTTGTAAGCGTACCGTGACATG	BIFC
GSA2-Fw	TACTAGTACAATGGCTGCGACGCTTAC	BIFC
GSA2-Rv	TCTCGAGGAGCCGACTTAGAACCTTC	BIFC
GBP-Fw	GCCCGGGAAAAATGCAACTCCAAACCCAATC	BIFC
GBP-Rv	CCCCGGGTTGTCCTCCTCCCTTGTG	BIFC
GluTR-Fw	GCCCGGGTTCAATGGCGGTTTCAAGTGCTTT	BIFC
GluTR-Rv	ACCCGGGCTTCTGTTGTTGTTCCGCG	BIFC

Supplemental Table S3. Primers used in this study. (Supports Figure 2-5.) The list of primers used for, genotyping the gsa1, gsa2 knockout mutants; cloning the *GSA1* genomic DNA in the pCAMBIA-Strep vector for the complementation constructs. The primers used for the transcript analysis for different TBS genes are indicated and the gene primers, which were used to fuse the N-terminal or the C-terminal half of pVyNE and pVyCE plasmids for BIFC analysis were also mentioned.

References:

Czarnecki O, Hedtke B, Melzer M, Rothbart M, Richter A, Schröter Y, Pfannschmidt T, Grimm B (2011) An Arabidopsis GluTR binding protein mediates spatial separation of 5-aminolevulinic acid synthesis in chloroplasts. Plant Cell **23**: 4476–4491.

Edgar RC, Drive RM, Valley M. (2004) Muscle: multiple sequence alignment with high accuracy and high throughput. Nucleic Acids Res **32**:1792–7.

Tamura K, Stecher G, Kumar S. (2021) MEGA 11: Molecular Evolutionary Genetics Analysis Version 11. Molecular Biology and Evolution.
