# Supplementary material for: Glutamate 1-semialdehyde aminotransferase is connected to GluTR by GluTR-binding protein and contributes to the rate-limiting step of 5-aminolevulinic acid synthesis
Source: Plant Cell. 2022 Aug 16;34(11):4623–40. doi: 10.1093/plcell/koac237 (PMC9614494; doi:10.1093/plcell/koac237)
Supplement: koac237_Supplementary_Data [file TPC2022RA00045R2_PeerReviewReport.pdf › koac237_Supplementary_Data/TPC2022RA00045R2_PeerReviewReport.pdf]

Glutamate 1-semialdehyde aminotransferase is connected to GluTR by GluTR-binding protein and contributes to the rate-limiting step of 5-aminolevulinic acid synthesis

Neha Sinha, Jürgen Eirich, Iris Finkemeier, Bernhard Grimm

Corresponding author: Bernhard Grimm, Bernhard.grimm@rz.hu-berlin.de

Review timeline:

**TPC2022-RA-00045**	Submission received:	Jan. 13, 2022
	1st Decision:	Feb. 15, 2022 *decline, might reconsider*
**TPC2022-RA-00045R1**	1st Revision received:	June 8, 2022
	2nd Decision:	July 6, 2022 *accept with minor revision*
**TPC2022-RA-00045R2**	2nd Revision received:	July 13, 2022
	3rd Decision:	July 15, 2022 *acceptance pending, sent to science editor*
	Final acceptance:	July 28, 2022

REPORT: (The report shows the major requests for revision and author responses. Minor comments for revision and miscellaneous correspondence are not included. The original format may not be reflected in this compilation, but the reviewer comments and author responses are not edited, except to correct minor typographical or spelling errors that could be a source of ambiguity.)

TPC2022-RA-00045 1st Editorial decision – *decline, might reconsider* **Feb. 15, 2022**

Thank you for choosing to send your manuscript entitled "Glutamate 1-semialdehyde aminotransferase is connected to GluTR by GBP and determines the rate-limiting ALA synthesis" for consideration at The Plant Cell. Your submission has been evaluated by four members of the editorial board as well as three expert reviewers in your field, and we regret to inform you that we are not able to recommend publication of this manuscript. We have not made this decision lightly. We have had input from multiple scientists, and have solicited post-review comments as well. Our present policy is to offer streamlined decisions and to not advise on the direction of the work by requesting extensive modifications or substantial additional experiments.

While there was an appreciation by the reviewers and editors for your multipronged approach to examine protein-protein interactions in the regulation of ALA synthesis, important concerns were raised from all reviewers that ultimately influenced our decision. Both reviewers 2 and 3 indicated that the claims are not entirely supported by the data and additional experimental evidence is required to make a compelling case for the conclusions. In addition, reviewer 1 suggested that the novelty of the conclusions is somewhat limited relative to what is already well established in the published literature across multiple organisms (including Chlamydomonas). Even if the issues raised by the reviewers 2 and 3 were to be addressed, the editors consider that this work would not represent a sufficient advance for us to be able to move forward in the direction of publication.

Response to appeal:

Although your email is not, strictly speaking, an appeal, I did go back to the editors to discuss your comments, the reviews, and the decision. You make a good point that we may have been overly harsh and reliant on the comments of Reviewer 1. With this in mind, we can change the decision to 'decline might reconsider'. I would reiterate that there are significant experimental concerns that were of equal if not greater significance to the novelty, in terms of their considering in making the earlier decision. That is, both Reviewers 2 and 3, with expertise in ALA metabolism, indicated that claims were not supported by the data, and while some or most of these issues may be addressable, they were numerous. But if you find them addressable as well, and are willing to provide the needed data, we would welcome a revision, and we would likely just go back to Reviewers 2 and 3 for their input.

-- Reviewer comments:

[Reviewer comments shown below along with author responses]

TPC2022-RA-00045R1 1st Revision received June 8, 2022

Reviewer comments and **author responses**:

Reviewer #2

This interesting work presents evidence that the two enzymatic steps of ALA biosynthesis, the committed steps of Tetrapyrrole biosynthesis for heme and chlorophyll, exist in a complex mediated by the "linker" protein GBP. They propose a reasonable and experimentally supported structurally model using x-linking data. This suggests that the GluTR enzyme alone does not dictate the levels of ALA accumulation as previously suspected. The evidence supporting the interactions among the three proteins is thorough and convincing, using several orthogonal approaches, while x-linking data provides nice support for a 3D structural model. There are, however, several points that must be addressed to clarify other experiments and convince me of other claims made in the text.

Major points:

1. page 8 lines 232-234 the authors claim a correlation between GSAAT activity and chlorophyll content. I am not sure of this as presented in the manuscript. First, it is not clear what measurement they use to gauge GSAAT activity, presumably the accumulation of ALA (Fig 4D) is the proxy measurement, but this is not clear. I guess this would be reasonable but the caveat would be that any subsequent use of ALA as substrate for downstream reaction would be assumed to be constant. Second, in Fig 4 panel E the chlorophyll measurements are presented, but which bar comes from CL-L, CL-M, and CL-H is not indicated. If I assume they follow the same sequence as ALA in panel D, then why does CL-M (the middle bar) show the highest ALA but the right bar (CL-H I think) in panel E is the highest, unless I am missing something, this doesn't support a correlation. This point must be clarified. I also suggest plotting a simple chart of data points to show the correlation between ALA ad chlorophyll as x and y -axes respectively, to provide simple demonstration of the correlation they claim. The claim is significant because it helps to support the idea that GSAAT activity is important for ALA and tetrapyrrole synthesis, not just GluTR.

> **Response: we wish to point out that the most relevant correlation exists in different light intensities between GSAAT content and ALA synthesis rate, which enables the production of more tetrapyrrole endproducts relative to increasing light intensities. In addition, an obvious correlation is detectable in LL AND ML conditions between GSAAT and Chl, while HL leads to a lower Chl content, likely due to adaptation of photosynthesis capacity and enhanced Chl turnover as result of repair of PSII subunits. According to the correct comment of the reviewer, the CL-L, CL-M, and CL-H were labelled in the revised figure 4.**

2. Figure 4, I don't see a labelled panel F, although it is referenced in the text. I assume the bottom right unlabeled panel is F?

> **Response: We regret that the label for panel F disappeared, but it can be seen again in Figure 4.**

3. page 8 lines 260-261 leaf samples were supplemented with ALA to test heme-dependent post-translational control of protein levels. Does ALA supplementation lead to heme accumulation? Perhaps this is a well known assay and the relationship has been demonstrated before, but this should be specified, otherwise please present the experimental evidence that the ALA supplementation produces heme accumulation.

> **Response: It is generally accepted that total heme content can hardly be increased, even in response to ALA supply. The excess of free heme is toxic and will be degraded. This is a common observation. Only a diminished ALA synthesis rate as result of a mutation in genes involved in ALA synthesis can be compensated by supply of ALA, which can lead to further accumulation of heme content up to wild-type levels. We performed the ALA feeding experiments in the background of gsa1 or gsa2 mutants,**

Minor points

1. I feel the Abstract can do a much better job of highlighting the key findings and their significance in light of prior understanding in the field.

Response: I followed the advice of the reviewer and entirely revised the abstract.

2. page 6 line 166. A description of the GSAAT antibody is necessary. Was it raised against one or the other isoforms (or both) and what part of the protein was used as epitope. And point out that the results from the two single mutants demonstrate it is reactive against both isoforms.

Response: The antibodies were raised against the recombinant barley GSAAT (Grimm, PNAS, 1990).

3. page 7 line 186 there is a higher content of GluTR and GBP levels in gsa2 mutant. Is it possible to provide a quantitative estimate of the levels? This was done for the Fig 4F, when levels of GSAAT were estimated as one-third of wild-type in gsa2 (page 8 line 226).

Response: We accept the comment of the reviewer and add quantification for the GSAAT, GluTR and GBP levels in figure panel 2D. Our estimation for the amount of GSAAT in Fig. 4F is approximately confirmed with one-third of wild-type content of GSAAT in gsa2.

4. page 7 line 198-200 claim the results indicate that "ALA synthesis primarily contributes to chlorophyll accumulation rather than heme synthesis" in young leaves. Possibly, but is it also possible this indicates the developing plant puts more importance on maintaining heme homeostasis? Perhaps these are not mutually exclusive possibilities?

Response: We agree with the reviewers' statement that at the same time control of heme homeostasis as well as predominately use of ALA for Chl synthesis is expected to happen. There is no disagreement. It was our intention to emphasize the higher quantitative demand of ALA for Chl synthesis than for heme synthesis. Heme is always required, most likely in more constant levels during development?

5. page 7 lines 212-213 the description of this light intensity experiment would benefit from some explanation of the rationale. What was the goal of the experiment and were the authors expecting a specific result?

Response: We observed a light intensity-dependent ALA synthesis rate. Apart from the known contribution of GluTR to rate-limiting ALA synthesis, we were interested to verify the impact of each GSAAT isoform on the control of ALA synthesis. As two genes encoding GSAAT are available, which is not common in the plant kingdom, we would like to assess the role of each isoform of GSAAT in leaves under different growth conditions.

We add additional comments about the rationales of these experiments described in this chapter.

6. Figure 5A, It would be nice if a higher magnification image could be provided, to see where in the cell the YFP is localizing. Is it overlapping with chlorophyll as expected?

Response: We followed the advice of the reviewer and the figure contains images with higher magnification. Scale bars were added in the figure panels of Fig. 6 and the legend informs about the size.

7. page 9 line 302, I don't "co-localized is the right word, should say something like "stably interacts".

Response: We follow this comment and intensified our efforts to carefully describe our results. We accept the more conclusive statement as suggested.

8. Figure 6 legend, doesn't describe the identity of the red ligands seen in the structural model. Also, a minor point but might help if each protein was colored with greater contrast colors, to help emphasize where each protein is found in the structure.

Response: Red ligand: The crystal structure of the GluTR GBP complex with the PDB identifier 5YJL was superimposed with a docking result based on GBP with Pymol. In the second step, the GSAAT structures after docking were superimposed upon GSAAT dimers from PDB structure 5HDM. Cofactors were derived from the respective crystal structures, and active-site residues of GluTR (Cys 144) and the Schiff-base-reactive Lys 274 of GSAAT (Song et al., 2016) are displayed as a red sphere The Superimposition of the heterodimeric complex comprising GluTR, GBP and GSAAT was more colorful displayed.

9. Discussion - I highly recommend starting the Discussion by discussing the most significant findings from the work. As it is, the Discussion starts with topic of the apparent equivalence of the two isoform, which is of interest to a much more specialized audience compared to the identification of the complex, or the significance of GSAAT in ALA synthesis rate. This relates to my general feeling that the significance of the work can be better drawn out in the Abstract and text.

Response: We acknowledge the reviewer's comment, but we prefer a discussion which starts with more marginal importance of aspects of the experimental work and ends with the main and highly emphasized issues. We are convinced that the manuscript benefits from this procedure. The discussion should end with the most significant aspects and the subsequent structural model.

10. page 13 line 455, the authors refer to "ALA-induced degradation of GluTR". Is this the same as the heme-inducaed degradation of GluTR (c.f. page 8 line 260) or different? Clarify in the text, and use consistent phrase if the same.

Response: Yes, we refer in the description to the initial experiments, which helped us to demonstrate the heme-dependent degradation of GluTR by ALA-feeding to leaves and the subsequently modified heme metabolism in chloroplasts.

11. The gsa2 mutant plant rosette size appears smaller, and then complemented (or over-compelemented?) by the GSAAT1 OE line. If this is a consistent observation, as it appears to be, the authors should include mention of this in the text.

Response: According to our observation, gsa2 mutants grow slower and have retarded growth and less rosette leaves biomass than the other genotypes, whereas the GSAAT1 OE lines grow faster and have more rosette biomass than the WT. We think, that the faster growth correlates with overproduction of GSAAT, und is not related to a specific deregulation of the isoform GSAAT1

12. Figure 2 legend - panel A is four week old or three week old. I think it is inconsistent between main text and figure legend. And is panel B measuring at 3 weeks, as for panel C?

Response: We revised the text and this issue is now consistently described: Four-week-old seedlings.

<u>Reviewer #3</u>

The manuscript "Glutamate-1-semialdehyde aminotransferase is connected to GluTR by GBP and determines the rate-limiting ALA synthesis" by Sinha et al. contains a significant amount of experimental work that seems mostly sound. However, the tone and style of writing do not meet the standards of The Plant Cell. Additionally, the evidence does not support the title from what I can discern. There is some evidence that GBP may be important in formation of a stable complex, but the claim that GSAT levels determine the rate of ALA synthesis seems to be based on mutant analysis. It is no surprise to anyone that loss of expression of an enzyme would lead to lower activity in the pathway. That does not make it rate-limiting. The interactions between these proteins is of some interest, but the claims made are unsupported by the evidence.

Response: we are confident to meet the standards of The Plant Cell. This applies also for the title. But we exchanged a verb indicating a more general phrase in the title of the manuscript. The main messages are emphasized with a key note. "Glutamate 1-semialdehyde aminotransferase is connected to GluTR by GBP and contributes the rate-limiting ALA synthesis"

Specific comments:

Line 34 "little is known" This seems to be an oversimplification of the knowledge available.

Response: We assume this is only a minor point. Compared to GluTR, very little is known about a regulatory impact of GSAAT to ALA and tetrapyrrole biosynthesis.

Line 43 "heterotrimeric protein complex" This is not even what the paper describes in the discussion. It is not a trimer, but a complex that may involve 8 polypeptides with homodimers of some of the enzymes.

Response: We follow the comment of the reviewer and for clarity we speak about an oligomeric complex.

Line 43 "scaffold protein" This term is used throughout the manuscript, but it does not seem accurate. Interaction does not make it a scaffold protein. In the model structure presented at the end, it seems like there is little if any evidence presented to support this and it is not clear what is even meant by this term.

Response: The term scaffold has been used in various connections and interdependencies. The function of scaffolding proteins is to bring together two or more proteins in a relatively stable configuration. Numerous scaffolding proteins are found in nature, many having multiple protein–protein interaction modules. https://www.molbiolcell.org/doi/10.1091/mbc.e14-04-0878

Lines 125-140. This paragraph is full of misstatements and attempts to use colloquial statements that are wrong. " At all events" was maybe supposed to be "in any event"? I would not use either in a scientific manuscript. The first sentence of the paragraph is also confusing

Response: In any case: Oxford English recommends the use of both terms with the same intention. We decided to omit the entire sentence.

Line 161- the diurnal oscillation of these genes does not necessarily correlate with synchronized enzyme activity. This seems to be a reach. Especially because in the next paragraph line 164 there is the observation that GSAAT levels remain constant. It is unclear whether there is turnover of GSAAT at all as suggested in line 166. The fact that GluTR oscillates suggests it is more likely to be the rate limiting part for ALA synthesis.

Response: yes, we agree with the reviewers view point and intent to phrase more cautiously. We agree with the general view that GluTR is considered to be the rate limiting enzyme. This follows the view on the previous publications, and our group had also a substantial share on these findings. However, we were able to complete the view on the ALA synthesis complex with this current report on GSAAT, which describes the contribution of GSAAT on the rate limiting step of ALA synthesis

Figure 2- are the bars on the graphs representing a range of values or standard deviation or standard error of the mean? How many replicates were used? For part C were statistics used to see if the ifferences between Wild type and mutant expression were significant?

Response: We acknowledge the suggestion of the reviewer and added information and statistics to the figures and legends?

Line 198 - Since the amount of heme relative to chlorophyll is quite low (picomoles versus micromoles) the statement in this sentence seems overly obvious.

Response: We accept the comment and modified the sentence in terms of the quantitatively different contribution of TBS metabolites for heme and chlorophyll accumulation. In the manuscript: "…but it is obvious that both GSAAT isoforms are required for an adequate ALA synthesis in chlorophyll-synthesizing leaves."

Line 202 Is it deletion of GSAAT2 or gsa2?

Response: It is an inactivation of GSA2 due to TDNA insertion. The mistake was corrected

Line 204 the connector "and" seems out of place here. Maybe make it 2 sentences?

Response: We disagree and suggest keeping the sentence.

Lines 223 and 224 have statements that are saying the exact opposite?

Response: To clarify this point, we rephrased the sentence.

Line 238 delete "however"

Response: We omitted the word.

Line 247-248 I strongly disagree with this conclusion. In the mutants it is limiting but that does not mean it is limiting in wild type. I also disagree with the statement that GSAAT defines anything. Wrong word.

Response: We accept that knock-out mutants cannot necessarily demonstrate a rate-limiting function of GSAAT to

ALA synthesis. But we keep in mind that the former conclusions of rate limiting function of GluTR also – at least partially -based on mutant analysis. We currently know something about the posttranslational control of GluTR, the different interacting factors affecting GluTR and ALA-synthesis activity and GluTR stability as well as the arrangement in stroma and membrane-associated fractions. Derived from these analyses, it becomes obvious that not the entire amount of GluTR contributes to ALA synthesis, but a certain amount is either inactivated or deposited. So far, the analysis of GSAAT contribution to ALA synthesis apart from their catalytic properties is just started. Our current knowledge on the quantitative contribution of GSAAT on ALA synthesis has just begun. GSAAT is only found in the soluble fraction of plastids. No aggregation or oligomerisation of GSAAT was observed. Thus, we feel the statement is reasonable that GSAAT contributes to the rate limiting capacities (line 247 of the paper).

Line 254 remove comma after Considering

Response: yes we corrected the sentence

Line 260 How were plants grown prior to ALA feeding? It seems like dark incubation might affect signaling and expression and make this experiment difficult to interpret.

Response: The dark treatment was performed as follows: Leaf discs of Arabidopsis Col-0, gsa1, gsa2 and 35S::GSA1 lines were transferred to buffer supplemented or not with 1 mM ALA. The water-soluble ALA is rapidly taken up through the leaf surface and metabolized in plastids, leading to substantially increased steady-state levels of Pchlide Side effects of reactive oxygen species (ROS) arising from accumulating tetrapyrroles were prevented by the dark incubation of detached leaves. Photoperiodic interference owing to diurnal variations in gene expression was excluded by first growing the plants in continuous light.

We think this protocol follows previous experiments performed by numerous groups working on TBS.

Line 325 it may suggest it facilitates interaction. I do not think it "characterizes GBP as a scaffold protein"

Response by definition: Wikipedia *https://en.wikipedia.org/wiki/Scaffold_protein* ...though scaffolds are not strictly defined in function, they are known to interact and/or bind with multiple members of a signalling pathway, tethering them into complexes…

We think the term is not only applicable in signaling. However, the use of this term is not absolutely necessary. At the request, we will exchange the term.

Line 338 The evidence presented is confusing. Intact chloroplasts were used? There still as an association in the pull down without GBP. The excess GBP level seems like it could be trapping without a specific interaction.

Response: We are not entirely clear what was meant. As result of the pulldown experiments of Supplemental Figure 5, the rather low amount of GSAAT from leaf extracts of the GBP-knock-out (KO) line compared to the amount of GSAAT pull-downed from extracts of the wild type or the GSA1-overexpressor lines is noteworthy. This result of GSAAT-pull down cannot be exclusively explained with the sole interaction to GBP. As we observe continuous ALA synthesis in the mutants lacking GBP, the ALA metabolism ensures functioning and efficient transfer of metabolites between GluTR and ALA and other unknown interacting factors are still plausible. The new included results of in vitro pull-down experiments with recombinant proteins demonstrate that GSAAT pull-down succeeds only with GluTR as bait by means of the assistance of GBP, but not in a direct interaction approach with GluTR (as bait) and GSAAT (Supplemental Figure 6).

Line 345 This pull down is not very convincing since the signal where it is supposed to demonstrate interaction is weak. The 6C figure has lanes that do not line up.

Response: We would show understanding for this comment, when Fig. 5C or Supplemental Figures 5 and 6 (but not Fig. 6C) are always the sole evidence for protein-protein interaction of the three proteins. The size fractionation of intact protein complexes is supported by additional studies to verify protein complex formation and protein-protein interactions as shown in this manuscript.

Line 365 What are "genomic signatures" ?

Response: The genomic signature refers to the characteristic frequency of oligonucleotides in a genome or sequence. It has been observed that the genomic signature of phylogenetically related genomes is similar. *https://en.wikipedia.org/wiki/Genomic_signature*. We have the impression this term is frequently used on issues in

genetics.

Line 383 I am not sure what you are trying to say here. They are isoforms so they catalyze the same reaction. That seems obvious.

Response. The potential difference of the isoforms are indicated in the next sentence of the paragraph.

Line 389 This may be an artifact of the overexpression. This is not well supported.

Response: We are not entirely sure what is meant with artefact. Overproduced GSAAT is found at the membrane, this is difficult to rebut. The consequences for membrane-associated GSAAT is currently only speculative.

Line 431 This is an over interpretation of the result

Response: As pointed out above, the current view on posttranslational control of ALA activity and stability as well as spatial arrangement of GluTR indicates that not the complete amount of GluTR always contribute to ALA synthesis.

Line 500 How were these relative polypeptide amounts determined? I do not see any experimental evidence to support it. Instead it seems like picking the number of units needed to fill the space in the model.

Response: The assessed molecular mass is deduced from encoded protein sequences.

Protein name	Transit peptide (amino acids)	Molecular wt. (Kd)	Mature protein (amino acids)	Molecular wt. (Kd)
GluTR	64	6.8	543	59.5
GSAAT 1	38	3.84	474	50
GSAAT 2	36	3.7	472	50
GBP	43	4.99	317	35.47

Line 596 What were the chloroplasts solubilized with? Multiple experiments were using chloroplast extracts. How were the chloroplasts isolated? I only see procedures described that would not create purified chloroplasts, but crude whole leaf extracts.

TPC2022-RA-00045R1 2nd Editorial decision – *accept with minor revision* July 6, 2022

We have received reviews of your manuscript entitled "Glutamate 1-semialdehyde aminotransferase is connected to GluTR by GBP and contributes to the rate-limiting ALA synthesis." On the basis of the advice received, the board of reviewing editors would like to accept your manuscript for publication in The Plant Cell. This acceptance is contingent on minor revision based on the comments of our reviewers. In particular, please consider the following:

The description of the MST experiments needs to be expanded for clarity. Also the labeling of "plastids" in Figures 5 (and Supplement) should be changed to avoid confusion-- perhaps "Isolated Plastids" or "Purified Plastids". Additional editorial corrections should be made as pointed out by the reviewers, and the manuscript would benefit from a thorough editing for word choice and grammar improvements.

TPC2022-RA-00045R2 2nd Revision received July 13, 2022

Reviewer comments and **author responses**:

Reviewer #2

The authors addressed my previous concerns satisfactorily. The MST is a nice touch that supports the interactions. I would point out a few points however:

Response: the paragraph about the experimental data obtained by MST was modified. I was done in the certainty of having provided more clarity.

1. The description of the MST experimental results in the Results section needs to be described better. As written it is difficult to discern which interactions are referred to according to the "gradually increasing" Kd and which number corresponds to which interaction.

Response: we refer to the previous sentence of our reply and to the modified paragraph describing the MST results. (last paragraph of the last chapter in the result section.

2. p13 lines 442-444, the sentence beginning "When GluTR is assembled ..." doesn't make sense to me, I think something is off grammatically.

Response: we revised the sentence and are confident that the revised sentences create more clarity about our intention.

3. p14 line 520 is it "inconsistent"? I think it is actually consistent.

Response: The term was revised.

Reviewer #3

This manuscript has been improved significantly by the inclusion of more detail and revision of the text. There are a few points that I think still need to be addressed to make things clearer for the reader. I think the work definitely provides evidence for a complex of proteins vis a variety of approaches and this complex is physiologically significant.

Major points

1. In both Figure 5 and Supplemental Figure 5 there is a heading for intact chloroplasts. In Figure 5B the first column is supposed to be actual intact plastids I think, but the second two columns are not intact chloroplasts because they doing pull down assays that would require the plastids to be lysed. The text describes these as extracts not intact plastids. This needs to be cleaned up. The same organization was used in Supplemental Figure 5. They added total plastid lysate, not intact chloroplasts from what I can tell

Response: The panels in figure 5 and supplemental figure were revised. We use the term plastid lysates.

Minor points.

1. line 168 says (see next chapter) I think they mean (see next section)

Response: According to our understanding. Chapter is correct, and as a compromise, we wrote next paragraph.

2. I am not sure how much the paragraph starting at line 398 adds to the manuscript. A proposal from 32 years ago before we gained much of the current knowledge regarding TBS does not seem so important to address. (This would allow the discussion to be condensed some).

Response: To emphasize the importance of this paragraph, this part of the manuscript was also revised, always with the intention to create more clarity and significance.

3. Line 412 refers to no specific evidence for the two forms of GSAAT having different biochemical characteristics. While technically true, the kinds of biochemical analysis to really determine this have not been done in this manuscript. Seems to be a bit of an overstatement.

Response: with the reference of the reviewer, the sentence was modified and hopefully clarifies our statement

4. I found the sentence in lines 442-44 very hard to understand and am still not sure what the point they were trying to make is.

Response: we revised the sentence and are confident that the revised sentences create more clarity about our intention.

5. Line 495 should indicate that the difficulty was in showing this relationship in plants. Other organisms had already shown a relationship of GluTR and GSAAT

Response: the reference to plants was added in the sentence.

TPC2022-RA-00045R2 3rd Editorial decision – *acceptance pending* **July 15, 2022**

We are pleased to inform you that your paper entitled "Glutamate 1-semialdehyde aminotransferase is connected to GluTR by GBP and contributes to the rate-limiting ALA synthesis" has been accepted for publication in The Plant Cell, pending a final editorial review by a science editor.

Final acceptance from Science Editor **July 28, 2022**
